# Size-Dependent Zinc Delivery and Nanozyme-Mediated Stress Alleviation by ZnO Nanoparticles in Zinc-Deficient Tomato

**DOI:** 10.1021/acs.jafc.6c02493

**Published:** 2026-10-10

**Authors:** Selahattin Kondak, Dóra Kondak, Tamás Bodor, Gábor Fejes, Attila Ördög, Andrea Rónavári, Zoltán Kónya, Réka Szőllősi, András Kukri, Attila Gyenesei, Péter Urbán, József Kun, Maria Gracheva, Ádám Solti, Kinga Benczúr, Magda Pál, Gabriella Szalai, Ildikó Domonkos, Titanilla Szögi, Fernando A. Casian Plaza, Gábor Galbács, Zsuzsanna Kolbert

**Affiliations:** † Department of Plant Biology, 37442 University of Szeged, Szeged 6726, Hungary; ‡ Doctoral School of Biology, Faculty of Science and Informatics, University of Szeged, Szeged 6726, Hungary; § Department of Applied and Environmental Chemistry, University of Szeged, Szeged 6720, Hungary; ∥ Hungarian Centre for Genomics and Bioinformatics, Szentágothai Research Centre, 37656 University of Pécs, Pécs 7624, Hungary; ⊥ Department of Pharmacology and Pharmacotherapy, Medical School University of Pécs, Pécs 7624, Hungary; # Budapest Neutron Centre, Institute for Energy Security and Environmental Safety, HUN-REN Centre for Energy Research, Budapest 1121, Hungary; ∇ Department of Plant Physiology and Molecular Plant Biology, Institute of Biology, ELTE Eötvös Loránd University, Budapest 1117, Hungary; ○ Agricultural Institute, 374774 HUN-REN Centre for Agricultural Research, Martonvásár 2462, Hungary; ◆ Institute of Plant Biology, HUN-REN Biological Research Centre, Szeged 6726, Hungary; ¶ Department of Pathology, Albert Szent-Györgyi Medical School, University of Szeged, Szeged 6720, Hungary; ⧅ Department of Molecular and Analytical Chemistry, University of Szeged, Szeged 6720, Hungary

**Keywords:** metabolomics, nanozyme, seed priming, suboptimal zinc supply, transcriptomics, zinc oxide nanoparticles

## Abstract

Zinc (Zn) deficiency severely limits plant growth and yield, highlighting the need for strategies to improve Zn nutrition. Here, Zn oxide nanoparticles (nZnOs) of two sizes (∼8 nm and ∼45 nm, 100 mg·L^–1^) were evaluated as seed priming agents in tomato grown under Zn-deficient conditions. Both nanoparticle sizes were internalized into embryonic tissues and showed distinct intracellular localization. While 45 nm nZnO and bulk Zn disturbed seed nutrient homeostasis, 8 nm nZnO increased seed Zn content more efficiently, with milder effects on other nutrients. Across developmental stages, nZnO priming improved Zn supply and nutrient distribution more effectively than bulk Zn. At the vegetative stage, 45 nm nZnO enhanced overall biomass, whereas 8 nm nZnO promoted root-specific growth under Zn deficiency. nZnO priming increased root Zn accumulation, particularly with 8 nm nZnO, and alleviated Zn deficiency-induced nonspecific metal accumulation. Zn deficiency-induced disturbances in redox and nitrosative balance were reversed by nZnO priming, accompanied by reduced RNA nitration and a shift toward a catalase-dominated hydrogen peroxide detoxification system. Both nanoparticle sizes exhibited peroxidase-like activity in cell-free assays, indicating nanozyme potential; however, this activity did not translate directly to the *in planta* responses. Under Zn deficiency, nZnO priming suppressed stress-related metabolic pathways in leaves, while enhancing amino acid, polyamine, and structural carbohydrate metabolism in roots. Zn deficiency-induced transcriptional repression, particularly in leaves, was partially restored, with affected genes enriched in stress-, phytohormone-, and detoxification-related pathways. Overall, size-dependent nZnO seed priming alleviates Zn deficiency in tomato. 45 nm nZnO mainly enhances biomass through improved nutrient homeostasis and redox regulation, while both sizes contribute to restored Zn availability and antioxidant reprogramming under Zn deficiency.

## Graphical abstract

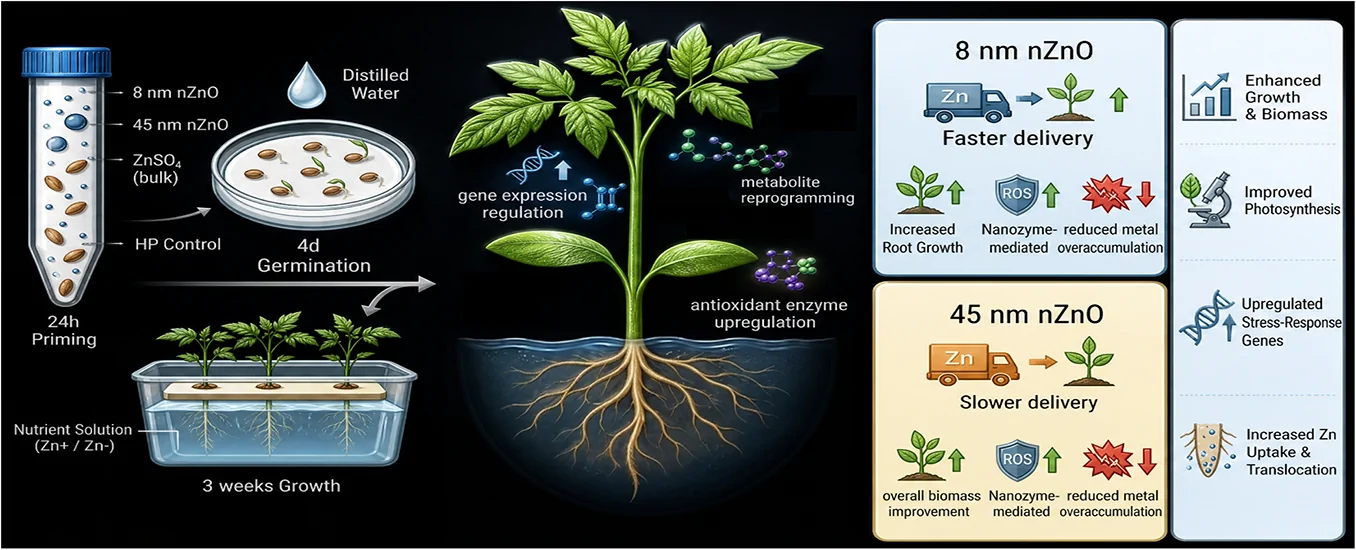

## 1 Introduction

Zn is an essential micronutrient that plays a central role in plant growth, development, and metabolic integrity. Although required in relatively small amounts, Zn acts as a cofactor for numerous enzymes and regulatory proteins and participates in key metabolic processes such as chlorophyll biosynthesis, carbohydrate metabolism, and gene expression.^1^ Zn is an integral component of metalloenzymes including carbonic anhydrase and Cu/Zn superoxide dismutase, which are crucial for photosynthetic carbon fixation and the detoxification of reactive oxygen species (ROS), thereby contributing to redox homeostasis.^1–4^ Beyond its metabolic functions, Zn influences protein stability and folding and is tightly linked to regulatory networks governing hormone balance and signal transduction. In particular, Zn’s involvement in auxin metabolism affects cell division, elongation, and differentiation, shaping root and shoot architecture during early growth and subsequent developmental stages.^4^ Therefore, suboptimal Zn availability commonly manifests in plants as stunted growth, reduced leaf expansion, chlorosis, and shortened internodes.^1^ Zn deficiency is among the most prevalent micronutrient constraints in agricultural soils worldwide, especially in alkaline, calcareous, and highly weathered soils where Zn bioavailability is limited by soil chemical properties and mineral interactions.^5^ Plants respond to low Zn supply through coordinated adaptive mechanisms involving enhanced Zn transport, chelation, and transcriptional reprogramming, as revealed by recent molecular and multilevel studies.^6,7^ Both Zn deficiency and excess harm plants—through oxidative stress, impaired photosynthesis, and disrupted micronutrient uptake—underscoring the need for precise Zn regulation to sustain optimal plant performance.^8^

Conventional Zn fertilization practices, including soil and foliar application of Zn salts, often suffer from low efficiency due to rapid immobilization in soil matrices, poor solubility under alkaline conditions, and restricted root uptake. As a result, a large proportion of the applied Zn remains unavailable to plants, necessitating repeated applications that increase economic costs and environmental burden.^9,10^ These limitations have stimulated interest in alternative approaches capable of improving Zn delivery and utilization. Over the past decade, nanotechnology has emerged as a promising strategy to address micronutrient inefficiencies by enabling targeted and controlled nutrient delivery at the nanoscale.^11^ Among nanoenabled formulations, zinc oxide (ZnO) nanoparticles (nZnOs) have received particular attention due to their high surface area, tunable physicochemical properties, and potential for enhanced interaction with plant tissues.^11–13^ Compared with conventional Zn sources, nano-Zn fertilizers have been reported to improve nutrient uptake, stimulate early growth, and reduce application rates while maintaining or enhancing agronomic performance.^14^ Limited understanding of nanomaterial behavior in the soil-plant-water system constrains their agricultural use;^15^ however, seed nanopriming—presowing treatment with minimal nanomaterial volumes—addresses this issue by avoiding large-scale field exposure. The behavior of nZnOs in plant systems is strongly influenced by their physicochemical properties, particularly particle size, which governs surface reactivity, dissolution kinetics, and interaction with biological barriers.^10,16^ Smaller nanoparticles often exhibit increased solubility and mobility, potentially facilitating faster Zn release and cellular access, whereas larger particles may function as slower-release Zn reservoirs, supporting sustained Zn availability during development.^17^ These size-dependent characteristics are especially relevant under Zn-deficient conditions, where efficient and timely Zn supply is critical for early growth and subsequent developmental processes. Accordingly, comparative assessments of differently sized ZnO nanoparticles provide an important framework for optimizing nanoenabled Zn delivery strategies.

Seed nanopriming has emerged as an effective approach to enhance seed performance and early seedling vigor by inducing physiological and metabolic adjustments prior to germination. In this context, nanoparticle-assisted seed priming offers a targeted means of delivering essential micronutrients directly to the seed, ensuring early-stage availability under nutrient-limiting conditions.^18,19^ ZnO nanoparticles applied during seed priming can interact with seed tissues, influence Zn localization during germination, and shape nutrient allocation during early seedling establishment. Such early exposure has been shown to affect redox balance, metabolic activity, and stress responsiveness, potentially priming plants for improved performance under Zn-deficient conditions.^20,21^

Plant responses to Zn availability and nanoenabled Zn delivery are also highly dependent on developmental stage. During early seedling growth, Zn demand is closely associated with cell division, root system establishment, and photosynthetic capacity, whereas later (vegetative) stages involve different physiological priorities and regulatory networks.^22^ The effects of ZnO nanoparticles observed shortly after germination may differ from those manifested during vegetative growth. Evaluating nano-Zn treatments across multiple developmental stages is therefore essential for capturing their dynamic influence on plant performance, particularly under Zn-deficient conditions.^16,23^

To specifically contextualize this work, the present study evaluated the effects of seed priming with differently sized nZnOs (∼8 nm and ∼45 nm) under Zn-sufficient and Zn-deficient conditions in tomato seeds, seedlings, and vegetative-stage plants. Tomato is considered a Zn deficiency-sensitive species, as inadequate Zn supply rapidly impairs its vegetative growth and induces characteristic symptoms, particularly under high pH or phosphorus-rich conditions. Seed priming was employed to ensure early-stage Zn availability and to investigate how nanoparticle size influences Zn uptake, growth performance, redox homeostasis, metabolic adjustment, and transcriptomic regulation. By integrating these multilevel analyses, the study aims to provide a comprehensive understanding of how differently sized nano-Zn treatments can enhance tomato adaptation to Zn deficiency, bridging general knowledge on nanoenabled Zn delivery with plant-specific physiological and molecular outcomes.

## 2 Materials and Methods

### 2.1 Synthesis of ZnO Nanoparticles

Two sizes of nZnOs were used in this study. Their synthesis and physicochemical characterization have been previously described in our earlier works,^24,25^ and the same batches were used in the present study. The smaller nZnOs (∼8 nm) were synthesized according to a modified method of Bian et al.^26^ Briefly, zinc acetate and potassium hydroxide were reacted in methanol under reflux at 60 °C, and the resulting precipitate was washed with acetone, dried, and ground into a fine powder. The larger nZnOs (∼45 nm) were prepared by precipitation of zinc chloride with ammonia solution, followed by washing, drying at 60 °C, and calcination at 450 °C for 2  h in air, as described by Srivastava et al.^27^ Detailed synthesis parameters and full physicochemical characterization (transmission electron microscopy, X-ray diffraction, ultraviolet–visible spectroscopy, and dynamic light scattering) are available in our earlier publications.^24,25^ To estimate Zn^2+^ release under the seed-priming conditions, suspensions of 8 and 45 nm ZnO nanoparticles (100 mg L^–1^) were incubated for 24 h under the same conditions used for seed priming. After incubation, nanoparticles were removed by centrifugation (60,000 g, 30 min) and filtration (0.22 μm), and dissolved Zn^2+^ in the supernatant was quantified by the Eriochrome Black T-based UV–vis assay.^81^

### 2.2 Plant Material, Growth Conditions, and Zn Deficiency Treatments

Seeds of tomato (*Solanum lycopersicum* L. cv. Folletto F1 (Manó)) were provided by Rédei Kertimag Zrt., Hungary. The seeds were surface-sterilized with 5% (v/v) sodium hypochlorite for 10 min. nZnO nanoparticles of two different sizes (∼8 nm and ∼45 nm) were used as seed priming agents at a concentration of 100 mg·L^–1^. For the nanoformulations, the particles were suspended in distilled water, initially forming a heterogeneous mixture containing large aggregates. The suspensions were sonicated for two cycles of 30 min to improve dispersion, and the pH was adjusted to 5.7–5.8. For the bulk treatment, ZnSO_4_ was dissolved in distilled water, with the pH similarly adjusted to 5.7–5.8, and the solution volume was set to achieve a final concentration of 100 mg·L^–1^ ZnSO_4_.

The seeds were primed in the respective nZnO or ZnSO_4_ solutions for 24 h in 15 mL Falcon tubes in the dark. After priming, the priming solution was removed, and seeds were briefly blotted on sterile filter paper to ensure that no priming solution remained. Seeds were then immediately transferred to Petri dishes containing filter paper moistened with distilled water and maintained in a growth chamber for 4 days. Following germination, seedlings were transferred to 1 L containers containing a complete tomato nutrient solution. For (Zn−) treatments, Zn was excluded from the nutrient solution, whereas for (Zn +) treatments, Zn was supplied as ZnSO_4_ from a 100 mM stock solution to achieve a final concentration of 2 μM. The basal nutrient solution contained the following macro- and micronutrients: 2 mM Ca­(NO_3_)_2_, 1 mM MgSO_4_, 0.5 mM KH_2_PO_4_, 0.5 mM Na_2_HPO_4_, 0.5 mM KCl, 10^–6^ M MnSO_4_, 10^–7^ M CuSO_4_, 10^–7^ M (NH_4_)_6_Mo_7_O_24_, 10^–7^ M AlCl_3_, 10^–7^ M CoCl_2_, 10^–5^ M H_3_BO_3_, and 2 × 10^–5^ M Fe­(III)-EDTA. Seedlings were grown for 3 weeks under controlled greenhouse conditions, including a photon flux density of 150 μmol m^–2^ s^–1^, a light/dark cycle of 12/12 h, relative humidity of 45–55%, and a temperature of 25 ± 2 °C.

### 2.3 Seed Stage Analysis: nZnO Internalization with Transmission Electron Microscopy (TEM) and Scanning Electron Microscopy (SEM)

All specimens were received in a 3% (w/v) glutaraldehyde solution at the Department of Pathology. Samples were postfixed in a fresh 3% (w/v) glutaraldehyde solution. After rinsing in phosphate-buffered saline (PBS), the samples were further fixed for 1 h in 2% (w/v) OsO_4_. After dehydration in increasing ethanol concentrations, the samples were rinsed in uranyl acetate and acetone. The samples were then embedded in EMbed 812 (Electron Microscopy Sciences, Hatfield, PA, USA). The embedded blocks were used to prepare ultrathin (70 nm) sections (Ultracut S ultramicrotome, Leica, Wetzlar, Germany), which were mounted on copper grids. The grids were counterstained with uranyl acetate (Merck, Germany) and lead citrate (Merck, Germany) and were examined and photographed with a JEOL JEM-1400 transmission electron microscope (JEOL, Tokyo, Japan). Representative micrographs were captured at magnifications of 2,500×, 6,000×, 12,000×, and 50,000× with the TEM Center software (JEOL, Tokyo, Japan).

For SEM measurements, the treated and dried seeds were mounted on SEM stubs, coated with a 15 nm layer of gold using a Quorum Q150T ES (Quorum Technologies, Lewes, UK) sputter coater, and imaged in a JEOL JSM-7100F/LV (JEOL SAS, Croissy-sur-Seine, France) scanning electron microscope at an accelerating voltage.

### 2.4 X-ray Fluorescence Imaging (XRF) Analysis of Elements in the Seeds

The distribution of elements was analyzed using XRF imaging. Seeds were dried at 60 °C to the air-dry stage and fixed onto thin window film for XRF imaging in a Horiba XGT-7200V system (Rh X-ray tube; Si drift detector). A standard Cu plate was used for energy calibration. XRF spectra were acquired under vacuum using a 100 μm-diameter X-ray guide tube (voltage: 50 kV; current: 1 mA). The distribution of Zn was analyzed by the characteristic Kα1 photons (peak energy of ∼8.616 keV). Distribution profiles were calculated by averaging the pixel values within defined regions of interest (ROIs) using the ImageJ Fiji software (v.2.16.0/1.54p with Java 1.8.0_322).

### 2.5 Seedling Stage Elemental Profiling by Laser-Induced Breakdown Spectroscopy (LIBS)

Prior to measurements, the seedlings were carefully removed from the Petri dishes and thoroughly washed with Milli-Q water to remove residual growth medium. Seedlings were then flat-pressed for 14 days using an object holder press (Kulzer GmbH, Germany), between layers of analytical filter paper sheets and polyethylene films to avoid any cross-contamination. Following drying, seedlings were embedded using a light-curing, one-component glycol methacrylate resin (Technovit 7200 VLC, Kulzer GmbH, Germany) onto 75 × 25 × 0.73 mm silicon substrates. In order to create a resin thin section with a level, smooth surface, a vacuum adhesive press (Exakt 402, Exakt Technologies, Germany) was employed. The use of 300 μm stainless steel spacers ensured a uniform section thickness.

LIBS analysis was performed using a J-200 LA/LIBS tandem instrument (Applied Spectra, Inc., USA) equipped with a 266 nm neodymium-doped yttrium aluminum garnet (Nd:YAG) laser emitting 6-ns pulses. The LIBS detection system consisted of a six-channel charge coupled device (CCD) spectrometer with a spectral window of 185–1050 nm and a resolution of 0.07 nm. LIBS elemental mapping was performed with a 0.5 μs gate delay, a 1.05 ms integration time, and a 10.0 mJ pulse energy. The sample surface area was mapped employing stepwise scans with 40 μm nonoverlapping laser spots. Embedded samples were ablated in seven layers. The number of layers was optimized by monitoring the Si emission signal; reaching a uniform, flat intensity across the mapped area indicated that the entire section was ablated. Analytical signals obtained from individual layers were accumulated to form a representative map of elemental distribution. Elemental maps were generated based on signals measured at the Zn I 472.2 nm, Ca II 393.3 nm, K I 766.5 nm, Mg I 618.4 nm, and Na I 588.9 nm emission lines, after background subtraction using the mean of 5 neighboring detector pixels for each emission line. LIBS measurements were controlled by the Axiom LA 2.4 operating software, and data processing was performed using ClarityNeX 1.0, both from Applied Spectra’s; elemental maps were acquired by the imaging software ImageLab 3.20 (Epina GmbH, Austria).

### 2.6 Biomass Production Assessment

The lengths of primary roots and shoots were measured manually and recorded in centimeters (cm). The fresh weight (FW) of roots and shoots was determined using an analytical balance, and the dry weight (DW) was determined after drying the plant material at 70 °C for 24 h. Both FW and DW were expressed in milligrams (mg). Additionally, leaf areas were measured using ImageJ software and recorded in square centimeters (cm^2^). Measurements were performed on fully expanded, mature leaves of intermediate size, avoiding both the youngest emerging and the oldest senescing leaves, from 3-week-old tomato seedlings.

### 2.7 Microelement Analysis by Inductively Coupled Plasma Mass Spectrometry (ICP-MS)

All ICP-MS measurements were performed using an Agilent 7700x ICP-MS instrument (Agilent, Santa Clara, USA) equipped with an Agilent I-AS autosampler unit. The plasma torch was operated at 15 L·min^–1^ Ar gas flow and 1550 W of radiofrequency (RF) forward power. The sample uptake rate, nebulizer gas flow rate, and sampling depth were set to 750 mL·min^–1^, 1.05 L·min^–1^ and 10 mm, respectively. The Agilent MassHunter instrument control and data acquisition software (Agilent, Santa Clara, USA) was used in all experiments. Data were recorded in analog mode, with an integration time of 1 s for all monitored masses (^11^B, ^23^Na, ^24^ Mg, ^39^K, ^44^Ca, ^45^Sc, ^55^ Mn, ^56^Fe, ^58^Ni, ^59^Co, ^63^Cu, ^66^Zn, ^78^Se) and tuning modes used. Signals for all isotopes were recorded in no-gas tune mode, except for ^44^Ca, ^56^Fe, and ^78^Se, for which the Agilent ORS3 collision cell (He mode) was used to reduce spectral interferences.

The roots and leaves of tomato plants were collected separately and subsequently dried at 70 °C for 24 h. A total of 100 mg of the dried tissue was subjected to microwave-assisted acid digestion (MarsXpress, CEM, Matthews, USA) using closed polytetrafluoroethylene (PTFE) vessels. Samples were digested at 200 °C and 1600 W for 2 h in a mixture consisting of trace-analytical-grade nitric acid (6 mL, 65% w/v; Reanal, Hungary) and hydrogen peroxide (2 mL, 30% w/v; VWR Chemicals, Hungary). Digested sample solutions were diluted by a factor of 83.3 and spiked with Sc (10 ppb) as an internal standard.

The ICP-MS was operated using high-purity Ar (99.996%) and He (99.999%) gases obtained from Messer Hungarogáz (Messer Hungarogáz Kft., Budapest, Hungary). All samples were prepared using trace-analytical-purity deionized water obtained from a Millipore Elix 10 device equipped with a Synergy polishing unit (Merck GmbH, Rahway, Germany). Standard solutions employed for calibration were prepared using a multielement stock solution (IV-ICPMS-71A, Inorganic Ventures, Christiansburg, USA). Elements used as internal standards were added to all solutions using a 6-element ICP-MS internal-standard stock solution (IV-ICPMS-71D, Inorganic Ventures, Christiansburg, USA).

### 2.8 Assessment of *In Planta* Antioxidant Enzyme Activities and Quantification of RNA Nitration

To assess the activity of key enzymatic antioxidants, including superoxide dismutase (SOD), catalase (CAT), and guaiacol peroxidase (POD), enzyme activities were measured spectrophotometrically using a V-730 spectrophotometer (JASCO, Tokyo, Japan) according to Tari et al.^28^ For enzyme extraction, 250 mg of fresh leaf tissue was ground in 1 mL of extraction buffer containing 100 mM phosphate buffer (pH 7.0), supplemented with 1 mM phenylmethylsulfonyl fluoride (PMSF) and 1% (w/v) polyvinylpolypyrrolidone (PVPP). The homogenate was centrifuged at 12000 g for 20 min at 4 °C. The resulting supernatant was collected into a microcentrifuge tube and used for subsequent enzymatic assays. SOD (EC 1.15.1.1) activity was evaluated based on the NBT color reduction assay, where one unit of SOD corresponds to a 50% inhibition of NBT reduction.^29^ CAT (EC 1.11.1.6) activity was determined by monitoring the decomposition of hydrogen peroxide (H_2_O_2_) at 240 nm, with one unit of CAT defined as the amount of enzyme that breaks down 1 μmol of H_2_O_2_ per minute.^30^ For ascorbate peroxidase (APX; EC 1.11.1.11), approximately 200 mg of frozen leaf tissue was homogenized in the presence of 1 mM ascorbate, followed by centrifugation at 12,000*g* for 20 min at 4 °C. APX activity was quantified by recording the decline in AsA concentration at 290 nm (ε 290 = 2.8 mM^–1^ cm^–1^) over a period of 1.5 min. One unit of APX is defined as the amount of enzyme required to oxidize 1 μmol AsA per minute.^28^ Soluble protein content was measured following the Bradford method,^31^ using bovine serum albumin (BSA) as the standard. POD (EC 1.11.1.7) activity was determined by following the increase in absorbance at 470 nm due to the oxidation of guaiacol (ε 470 = 26.6 mM^–1^ cm^–1^). The amount of enzyme catalyzing the formation of 1 μmol min^–1^ 30 of oxidized guaiacol was defined as 1 U.^32^

RNA nitration levels were quantified using the EpiQuik Nitrosative (8-Nitroguanine) DNA/RNA Damage Quantification Kit (Colorimetric), following the manufacturer’s instructions. Total RNA used in this assay was extracted as described in Section 2.12. Although the kit is designed for detecting damage to both DNA and RNA, only RNA samples were analyzed in this study. This assay is based on the specific recognition of 8-nitroguanine (8-NG in RNA is a modified nucleobase formed by reactive nitrogen species during stress responses), a nitrosative stress-derived nucleic acid modification, and enables sensitive detection of nitrated RNA in biological samples. Both leaf and root tissues were processed, and extracts were prepared according to the kit protocol, loaded into antibody-coated wells, and incubated with the detection complex. The colorimetric signal was measured at the manufacturer’s recommended wavelength, and 8-nitroguanine levels were calculated from the standard curve provided in the kit. 8-NG levels were quantified as a percentage of total RNA (8-NG%) of total RNA, representing the proportion of nitrated nucleobases relative to total RNA content, and were calculated according to the manufacturer’s formula using optical density values normalized to positive and negative controls.

### 2.9 *Ex Planta* Peroxidase (POD)-Like Activity of the nZnOs

The peroxidase-mimicking activity of the nZnOs was evaluated as previously described,^33^ using H_2_O_2_ and 3,3’,5,5-tetramethylbenzidine (TMB) as substrates. For the assay, 200  μL reaction mixtures were prepared containing TMB  (6  mM) and nZnOs (0.5 mg·mL^–1^) in 100 mM acetate buffer (pH 4.0). Control reactions were prepared under identical conditions without nZnOs. The reaction was initiated by the addition of H_2_O_2_ at 37 °C, and the formation of the oxidized TMB product was measured by UV–visible spectroscopy in the range of 400–750 nm.

### 2.10 Fluorescence Imaging of Reactive Nitrogen and Oxygen Species, Intracellular Zn and Cell Viability

Semiquantitative detection of NO and ONOO^–^ in the tomato root tips was carried out using fluorescent probe-based staining.

To visualize NO, the samples were treated with 10 μM DAF-FM DA (4-amino-5-methylamino-2′,7′-difluorofluorescein diacetate). The dye was dissolved in 10 mM Tris-HCl buffer (pH 7.4), and root tissues were incubated for 30 min at room temperature in the dark. After incubation, the samples were rinsed twice with the same buffer and subsequently mounted on slides for imaging.^34^

For ONOO^–^ determination, 10 μM aminophenyl fluorescein (APF) was used. Root samples were incubated with the APF solution for 1 h at room temperature in the dark conditions. Afterward, they were washed twice with 5 mM Tris-HCl buffer (pH 7.4) and prepared for microscopy.^34^

Time-dependent monitoring of Zn-ion accumulation in tomato root tips was performed using the ethyl (2-methyl-8-*p*-toluenesulfonamide-6-quinolyloxy)­acetate fluorescent probe (Zinquin), following the procedure described by Helmersson et al.^35^ Root tips were first equilibrated in PBS, after which they were incubated in a 25 μM Zinquin solution prepared in PBS for 60 min at room temperature in the dark conditions. Prior to microscopic observation, the samples were rinsed once with PBS. Zinquin-derived fluorescence was examined using an epifluorescence microscope.

H_2_O_2_ accumulation was assessed using 50 μM Amplex Red (10-acetyl-3,7-dihydroxyphenoxazine), dissolved in 50 mM sodium phosphate buffer (pH 7.5). The staining was performed by incubating root tips in the dye solution for 30 min at room temperature in the dark, followed by rinsing in the same sodium phosphate buffer before slide mounting.^36^

Superoxide anion radical (•O_2_^–^) levels were visualized with dihydroethidium (DHE). Root tips were immersed in a 10 μM DHE solution prepared in 10 mM Tris-HCl buffer (pH 7.4) and incubated for 30 min at 37 °C in the dark. Afterward, the samples were washed twice with the buffer and transferred to slides for microscopic examination.^34^

Root meristem cell viability was determined using fluorescein diacetate (FDA) staining. Tomato root tips were incubated in 10 μM FDA solution for 30 min at room temperature in the dark, then washed twice with 10 mM Tris-HCl buffer (pH 7.4), following the protocol of Kolbert et al.^34^

All fluorescence analyses were performed on a Zeiss Axiovert 200 M microscope (Carl Zeiss, Jena, Germany) equipped with an Axiocam HR digital camera and 10× objectives for root samples. Filter sets were applied as follows: Filter set 10 (exc. 450–490 nm, em. 515–565 nm) for DAF-FM and APF, Filter set 9 (exc. 450–490 nm, em. 515–∞ nm) for DHE; Filter set 20 (exc. ∼540 nm, em. ∼590 nm) for Amplex Red; and the DAPI filter set (exc. 365 nm, em. 430–480 nm) for Zinquin.

Pixel intensity measurements were obtained using AxioVision Rel. 4.8 software (Carl Zeiss, Jena, Germany). Circular measurement areas had a diameter of 120 μm for root tips. Each analysis was repeated at least three times, evaluating 10 root tips (n = 10).

### 2.11 Chlorophyll a+b Quantification

Chlorophyll a+b content was quantified following the procedure described by Yudiansyah and Aziz.^37^ Fresh leaf samples were homogenized in an organic solvent (100% acetone) and then incubated at 4 °C for 24 h. Samples were transferred to 2 mL Eppendorf tubes and further extracted with 80% acetone prepared using 10 mM Tris buffer (pH 7.8) according to the referenced protocol, and the extracts were clarified by centrifugation (22,540*g* for 15 min at 4 °C). The absorbance of the supernatant was then measured spectrophotometrically (V-730, JASCO, Tokyo, Japan) at wavelengths (470, 537, 647, and 663 nm (Sims and Gamon, 2002)) characteristic for chlorophyll a and chlorophyll b, and total chlorophyll content (a+b) was calculated using the extinction coefficients provided in the method. This approach enables accurate estimation of chlorophyll levels and is widely used for assessing photosynthetic pigment status under different physiological conditions.

Pigment equation formula using the Sims and Gamon equation:^38^

$$Chlorophylla(\mu mol\cdot mL^{-1})=0.01373A663-0.000897A537-0.003046A647$$

$$Chlorophyllb(\mu mol\cdot mL^{-1})=0.02405A647-0.004305A537-0.005507A663$$

### 2.12 Transcriptome and Bioinformatics Analysis

Approximately 100 mg of tissue per sample was flash-frozen in liquid nitrogen and stored at −80 °C until processing. Total RNA extraction was first performed using a Zymo Research RNA purification kit to remove potential contaminants and inhibitors. Following total RNA extraction, the samples were subjected to an additional purification step to ensure high-quality RNA suitable for downstream analyses. The isolated RNA was purified using the E.Z.N.A. MicroElute RNA Clean-Up Kit (Omega Biotek, Inc., Norcross, GA, USA). RNA libraries were generated using the QuantSeq 3′ mRNA-Seq V2 Library Prep Kit FWD with UDI (Lexogen, Vienna, Austria). Briefly, 100 ng of total RNA was used as input for first-strand cDNA generation using an oligo­(dT) primer, followed by removal of the RNA removing. Thereafter, second-strand synthesis was initiated by random priming, and the products were purified with magnetic beads. Finally, the libraries were amplified and barcoded using PCR. All libraries were assessed on the Agilent 4200 TapeStation (Agilent Technologies, Palo Alto, CA, USA) to examine if adapter dimers formed during PCR. The QuantSeq libraries were sequenced using the Illumina NovaSeq X Plus platform (Illumina, San Diego, CA, USA) to produce 75 bp single-end reads.

Binary Base Call (BCL) files generated by the sequencing instrument were base-called, demultiplexed, and translated into FASTQ files using BCL Convert software v4.2.7 (Illumina, Inc.). Reads were filtered for a minimum length of 40 bp and quality-trimmed to Q30 using the Phred algorithm with BBDuk (BBTools suite v38.86, sourceforge.net/projects/bbmap/) then aligned against the tomato genome (SLM_r2.1, NCBI RefSeq assembly GCF_036512215.1). Reads aligned within each gene were counted using HTSeq v0.11.1.^39^ The Nextflow workflow manager was used to orchestrate these steps.^40^ The nf-core/differentialabundance pipeline v1.5.0 ^41^ was used to carry out gene count data processing and differential expression analysis, applying the DESeq2 R package.^42^ Gene Ontology (GO) overrepresentation analysis (ORA) was performed using the shinyGO v0.85 web platform (https://bioinformatics.sdstate.edu/go/).^43^ ORA and gene set enrichment (GSEA) for Kyoto Encyclopedia of Genes and Genomes (KEGG) pathways were performed using clusterProfiler v4.12.6 ^44^ gage v2.36.0 ^45^ and pathview v1.26.0 ^46^ packages in the R v4.4.0 environment, with the hypergeometric test and the nonparametric Kolmogorov–Smirnov test for ORA and GSEA, respectively.

### 2.13 Quantification of Polyamines, Amino Acids, Organic Acids, and Sugars

For polyamine (PA) analysis, 200 mg of plant tissue was homogenized with liquid N_2_ in a mortar and further ground with 2 mL of 0.2 N HClO_4_. The homogenates were centrifuged at 4 °C for 10 min at 10,000*g*. For dansyl chloride derivatization reactions, 100 μL of supernatant and 200 μL of saturated sodium carbonate were used according to Németh et al.^47^ where the reaction was started with 400 μL of dansyl chloride in acetone (5 mg·mL^–1^). The mixture was incubated at 60 °C for 1 h in the dark, and stopped with 100 μL of proline (100 mg·mL^–1^). After further incubation at room temperature in the dark for 30 min, the PAs were extracted by vigorous vortexing with 500 μL of toluene for 30 s. The upper, organic phase containing the PAs was removed and completely dried. The residue was dissolved in 0.5 mL of methanol, ultrafiltered through nylon membranes, and used for high performance liquid chromatography (HPLC) analysis. The analysis of dansylated PAs, namely, 1,3-diaminopropane (DAP), putrescine (PUT), spermidine (SPD), and spermine (SPM), was carried out according to Pál et al.^48^ via HPLC using a W2690 separation module on a reversed-phase column (Kinetex C18, 5 μm, 100 × 4.6 mm, Phenomenex, Inc.) and a W474 scanning fluorescence detector with excitation at 340 nm and emission at 515 nm (Waters, Milford, MA, USA).

Samples were extracted for sugar and organic acid analysis. Sample preparation was based on Gondor et al.,^49^ with minor modifications. Samples were extracted twice with 60% methanol and twice with 90% methanol after the addition of the internal standard (ribitol, 1 mg·mL^–1^). An aliquot of the supernatant was dried prior to derivatization. The QP-2010 GC-MS instrument (Shimadzu Corp., Kyoto, Japan) was used in split mode. The thermal program and separation phases followed those described in Gondor et al.^49^ Metabolite detection was performed using standards and the Kovats retention index. For gas chromatography (GC) analyses, data evaluation and metabolite normalization were carried out using the LabSolution (GCMSolution version 4.45, Shimadzu Corp.) software. Data processing was performed with LabSolution Postrun (GCMSolution version 4.45, Shimadzu Corp.), the NIST 2.3 and Wiley libraries.

### 2.14 Statistical Analysis

Results are presented as means of the raw data, with corresponding standard deviations or standard errors (±SD or ±SE). Statistical analyses were performed in SigmaPlot version 12 using Student’s *t*-test or Duncan’s multiple range test following one-way ANOVA (*P* < 0.05. Prior to ANOVA, homogeneity of variances was assessed using Hartley’s *F*_max_ test, and data normality was evaluated with the Shapiro-Wilk test.

## 3 Results

### 3.1 Characterization of ZnO Nanoparticles

Physicochemical properties of the two nZnO particle types used in this study were previously characterized in our earlier works.^24,25^ The same nanoparticle batches were used in the present study without further modification. Both nZnOs were quasi-spherical in shape; the smaller nZnO particles exhibited an average diameter of approximately 8 nm, whereas the larger nZnOs were ∼45 nm in size. The chemical composition and crystallinity of both samples were verified by electron diffraction and X-ray diffraction analyses.

### 3.2 nZnO Internalization into Seeds

Since the tomato is a member of the Solanaceae family (subfamily *Solanoideae*), its seed structure exhibits characteristic features. The seed contains an embryo composed of a plumule, a radicle, and two cotyledons, which is surrounded by a cellular endosperm and a hairy seed coat. The embryo is flat and curved, and the tip of the radicle is protected by a micropylar cap, a structure typical of Solanoideae-type seeds. This cap represents the remnant of the ovule micropyle and marks the site where the radicle emerges during germination. The micropylar pore also serves as the entry point for water and water-soluble nanoparticles into the seed^50^ (Figure 1A,B). In tomato seeds primed with 8 nm nZnO, TEM demonstrated nanoparticle uptake and incorporation into embryonic cells (Figure 1C). Rather than discrete, round particles, smaller nanoparticles were observed in clustered forms. These irregularly shaped aggregates were found in vesicle-like, electron-lucent compartments (Figure 1D). Uptake of 45 nm nZnO during the priming process and subsequent incorporation into embryonic cells were also detected (Figure 1E,F). In cotyledons, globular electron-dense particles with sizes close to 45 nm were observed within lipid bodies (Figure 1F). Light microscopic observations of toluidine blue-stained sections revealed that tomato cotyledon cells contain abundant vesicles corresponding to lipid (oil) bodies^51^ (Figure 1B).

**Figure 1:**
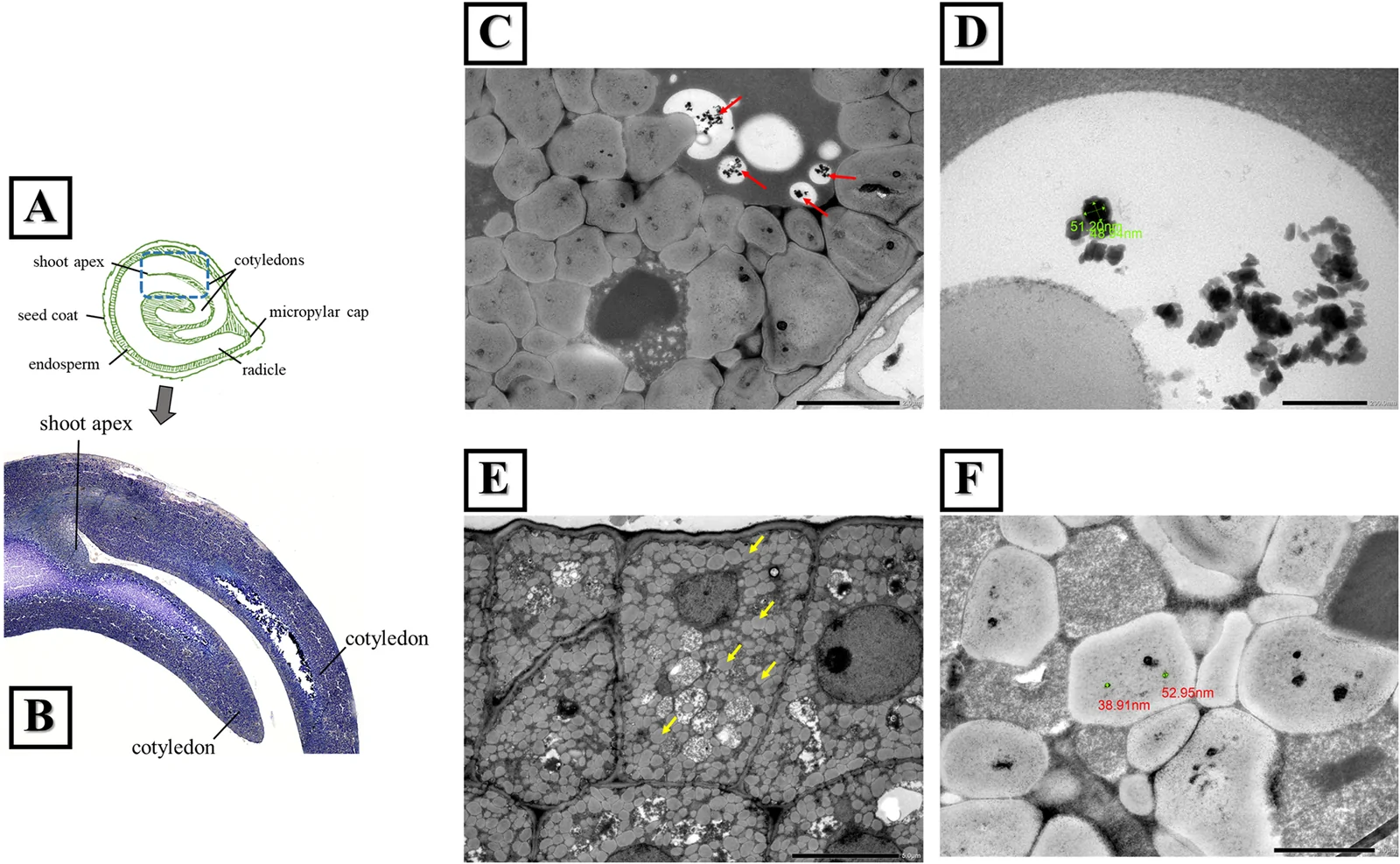
Schematic structure of a mature tomato seed with a dicot embryo (A), and the longitudinal section of a tomato embryo stained with toluidine blue (B) (http://www.seedbiology.de/structure.asp). Transmission electron microscopic (TEM) micrographs of tomato cotyledons derived from 8 nm nZnO-primed seeds at 6,000× magnification, where red arrows indicate probable nanoparticle aggregations (C), and at 50,000× magnification, where the sizes of two representative aggregations are shown (D). TEM micrographs of tomato cotyledons derived from 45 nm nZnO-primed seeds at 2,500× magnification, where yellow arrows indicate intracellular lipid bodies (E), and at 12,000× magnification, where the sizes of two representative globular particles are shown (F). Scale bars = C: 2 μm; D: 200 nm; E: 5 μm; F: 1 μm.

Moreover, SEM analysis showed that the seed coat remained intact in both hydroprimed control seeds and nZnO-primed seeds, displaying a continuous surface without fissures, depressions, or collapsed regions. At the same time, 8-nm nZnO priming resulted in seeds with reduced hairiness compared to 45-nm nZnO, where the seed trichomes seemed to be intact (Figure S2).

### 3.3 Impact of Seed Stage Treatments on Tissue Elemental Profiles

The elemental profiles of hydroprimed, nZnO-primed, and bulk Zn-primed tomato seeds were compared by μXRF. Zn levels were induced most strongly by 8 nm nZnO (∼470%), and 45 nm nZnO caused similarly high Zn accumulation (∼360%) in the seed, while bulk Zn priming led to significantly lower Zn accumulation (∼130%). Zn associated with nZnO - or bulk Zn accumulates predominantly in the outer seed layers (Figure 2A), whereas the embryo exhibits a relatively lower Zn supply, as supported by the negative difference between the normalized Zn content of the inner seed tissues and that of the whole seed for Zn and other elements (Figure S3, S4).

**Figure 2:**
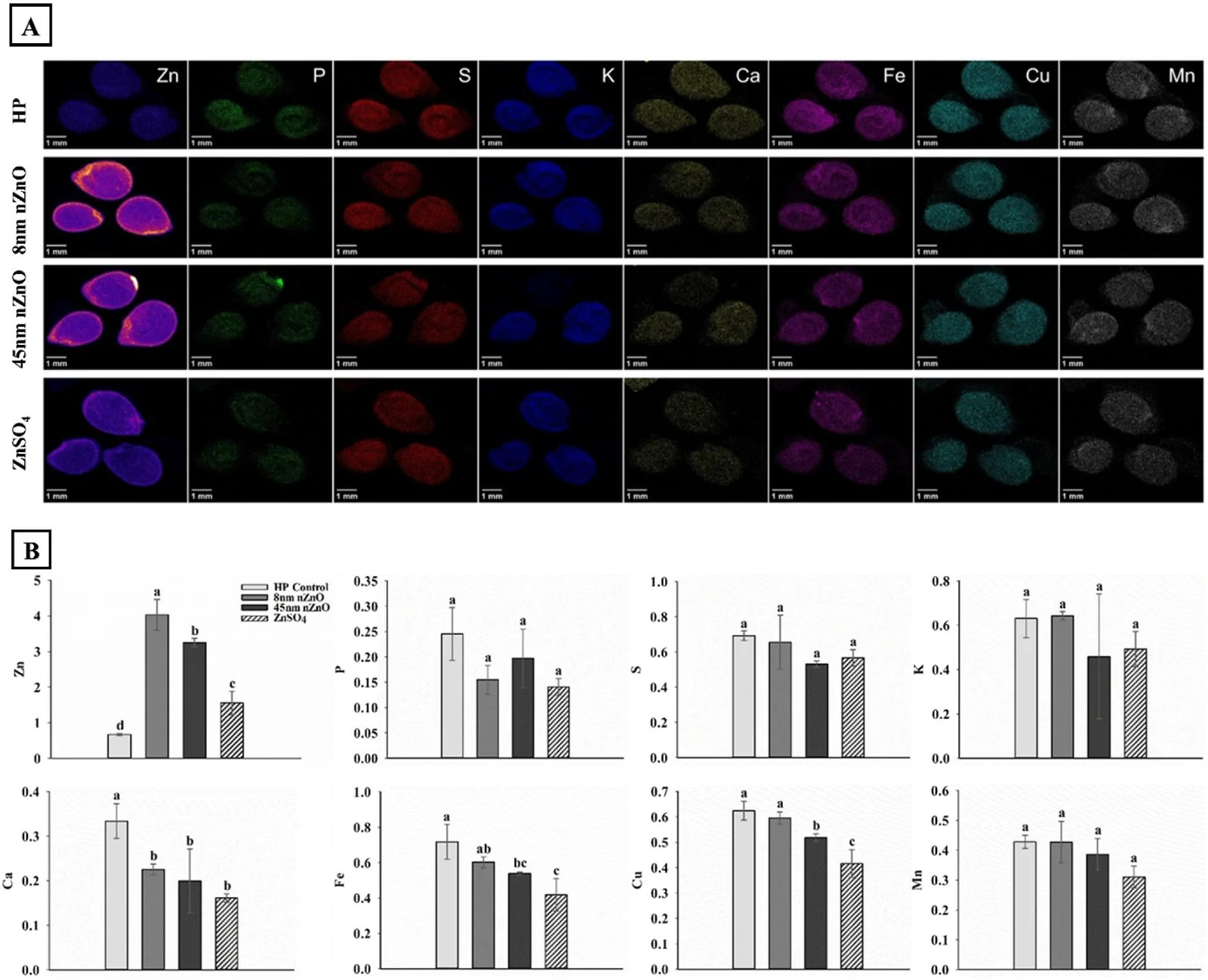
μ-XRF maps of tomato seeds showing element distribution. Scale bars = 1 mm (A). Normalized contents of different elements measured in whole seeds of tomato (B). The seeds were primed for 24 h with distilled water (HP), 8 nm nZnOs (100 mg·L^–1^), 45 nm nZnOs (100 mg·L^–1^), or ZnSO_4_ (100 mg·L^–1^). Different letters indicate statistically significant differences according to Duncan’s test (*p* < 0.05).

Seed priming with 45-nm nZnO or bulk Zn had the most significant effect on seed element distribution, as it significantly reduced Ca, Fe, and Cu levels and resulted in statistically nonsignificant reductions in P, S, K, and Mn levels. The 8-nm nZnO reduced only Ca levels, while the other elements were not significantly modified (Figure 2A,B).

To evaluate the contribution of dissolved Zn during seed priming, dissolved Zn^2+^ was quantified after a 24 h incubation under the priming conditions. Approximately 38% of the total Zn was released from the 8-nm nZnO, whereas ∼17% was released from the 45-nm nZnO, indicating substantially greater dissolution of the smaller nanoparticles during the priming period.

### 3.4 Element Composition in nZnO-Primed Seedlings

As a next step, we used LIBS to investigate the distribution of certain nutrient cations in seedlings developing from primed seeds (Figure 3).

**Figure 3:**
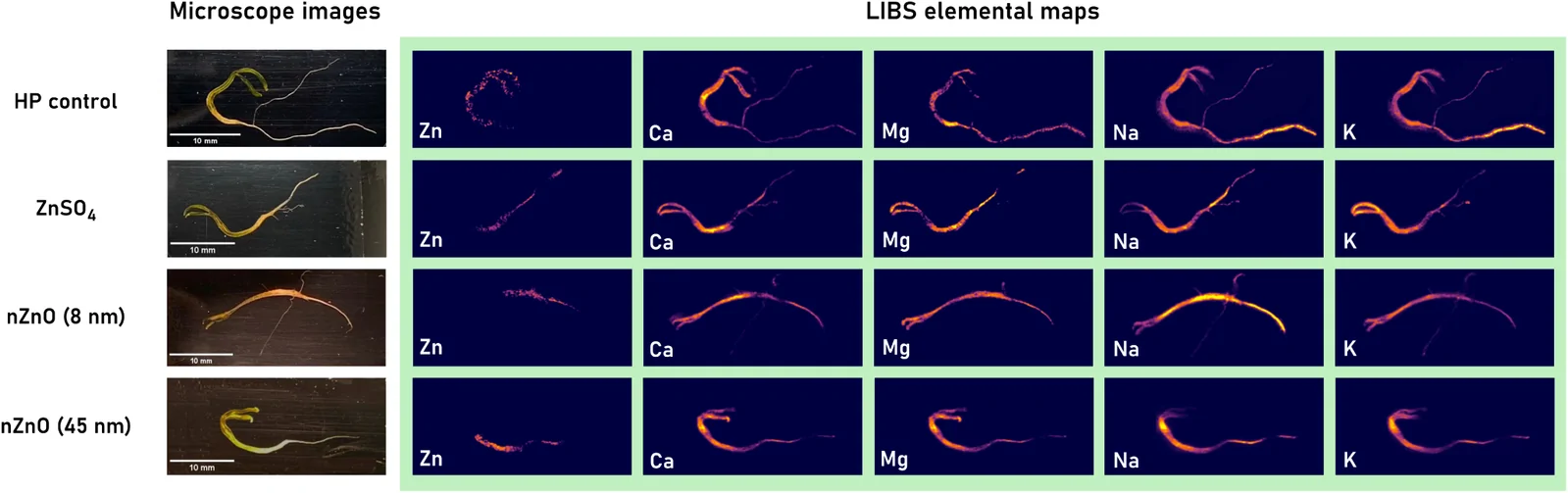
Representative LIBS elemental maps demonstrating the *in planta* distribution of selected macro- and microelements in 12-days-old-tomato seedlings grown from seeds primed with distilled water (HP control), 8 nm nZnOs (100 mg·L^–1^), 45 nm nZnOs (100 mg·L^–1^), or ZnSO_4_ (100 mg·L^–1^). The color shading in the maps corresponds to the net optical emission intensity of the given spectral line of the element, which is proportional to the concentration (e.g., dark blue corresponds to a very low concentration, and bright yellow corresponds to a high concentration). Scale bars = 10 mm.

In the case of seedlings grown from hydroprimed seeds, only the hypocotyl and cotyledon show a baseline Zn LIBS signal. As a result of 45 nm nZnO seed priming, the Zn signal in the hypocotyl and in the part of the root closer to the shoot base slightly increased. Compared to Zn, Na was more readily detectable in tomato seedlings, and 8 nm nZnO caused an increase in the Na signal in the hypocotyl and root, while 45 nm nZnO seed priming increased it in the hypocotyl and decreased it in the root. Additionally, bulk Zn priming resulted in unchanged Na levels in the hypocotyl and decreased Na levels in the seedlings’ roots. The calcium signal in the root increased more with 8 nm nZnO than with 45 nm nZnO or bulk Zn seed priming; the latter treatments increased Ca levels primarily in the root part close to the shoot base. Magnesium levels were increased in the hypocotyl and decreased in the root mainly by 45 nm nZnO and bulk Zn seed priming (Figure 3).

### 3.5 Biomass Production and Growth Responses to Treatment with Different-Sized nZnOs in Zn-Deficient Tomato

Under optimal Zn supply, seed priming with 8 or 45 nm nZnO did not significantly modify the measured root and shoot parameters compared to hydroprimed control plants (Figure 4); however, seed priming with the same concentration of bulk Zn (ZnSO_4_) significantly decreased shoot fresh and dry weights (Figure 4B,C). This indicates that shoot biomass production is notably sensitive to Zn excess provided through seed treatment. Suboptimal Zn supply significantly reduced shoot biomass parameters (shoot length, fresh and dry weights, leaf area) in tomato (Figure 4A–D and H), while in the case of roots, the length (Figure 4E,H) and dry weight (Figure 4G,H) were not significantly modified compared to the full Zn condition. Additionally, root fresh weight was decreased by ∼75% compared to plants with adequate Zn supply (Figure 4F and H). Overall, these findings indicate that tomato is sensitive to inadequate Zn supply, the shoot is more sensitive to Zn deficiency than the root system, and root water uptake is significantly reduced. Seed priming with the smaller nZnO (∼8 nm) significantly improved root fresh and dry weights (Figure 4F,G,H), while shoot biomass parameters and root length of Zn-deficient plants were not significantly increased (Figure 4A–D,E,H). In contrast, the application of larger-sized nZnO as a seed priming agent significantly improved shoot fresh weight, leaf area, and root fresh and dry weights in Zn-deficient tomato (Figure 4B,D,F,G,H). Seed priming with bulk Zn had a positive effect only on shoot length (Figure 4A), but the other measured parameters were not significantly improved (Figure 4B–H).

**Figure 4:**
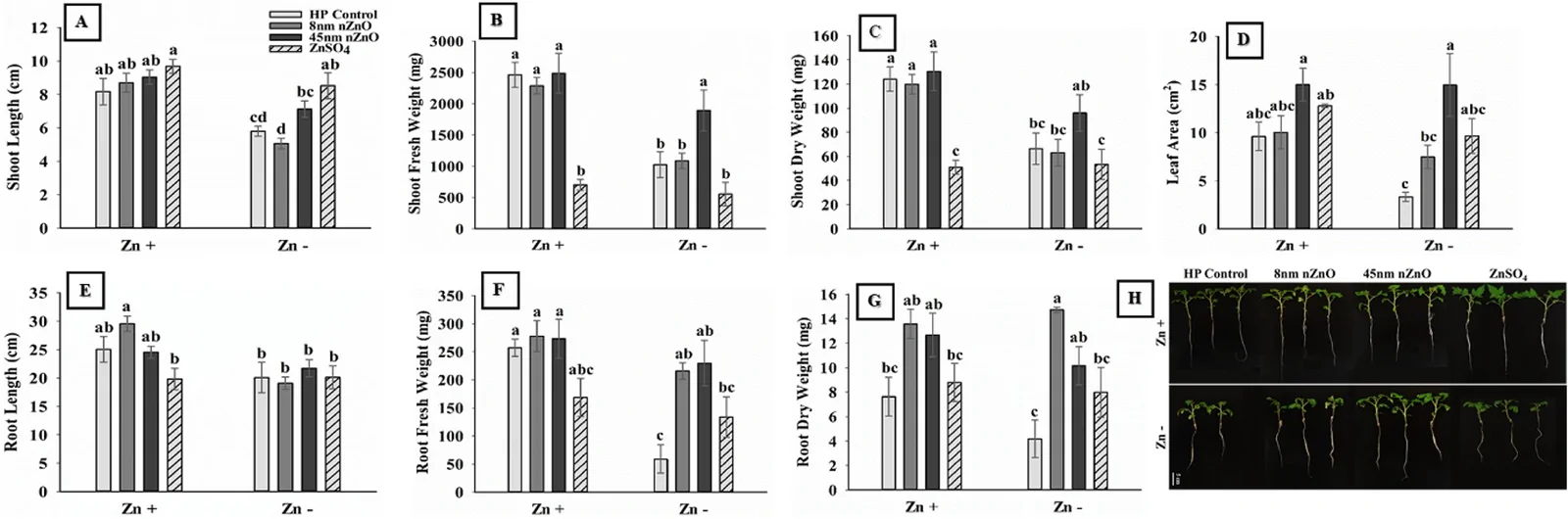
Biomass production (shoot length, A; shoot fresh weight, B; shoot dry weight, C; leaf area, D; root length, E; root fresh weight, F; root dry weight, G) of vegetative-stage tomato plants grown with optimal (Zn +) or suboptimal (Zn−) Zn supply. Tomato seeds were primed with distilled water (HP control), 8 nm nZnO (100 mg·L^–1^), 45 nm nZnO (100 mg·L^–1^) or ZnSO4 (100 mg·L^–1^). Different letters indicate statistically significant differences according to Duncan’s test (*p* < 0.05). (H) Representative photographs showing three-week-old tomato plants grown from primed seeds and cultivated with optimal or suboptimal Zn supply. Scale bar= 5 cm.

### 3.6 Zn and Other Microelement Concentrations

The significant increase in biomass may be partly due to the release of Zn ions from nZnOs; therefore, the concentrations of Zn and other microelements were measured in the root and shoot systems of tomato.

Zn deficiency induced a characteristic elemental imbalance in both roots and shoots. As expected, Zn concentrations declined in both leaves and roots of tomato plants grown from hydroprimed seeds and cultivated under suboptimal Zn supply, and this reduction was accompanied by decreased Cu levels. In contrast, Fe, Mn, and Co contents increased in both organs, while B remained unchanged in plants grown under inadequate Zn supply compared to plants cultivated with full Zn supply. Ni exhibited a distinct pattern, showing a Zn deficiency-induced increase specifically in the root. Seed priming with nZnO produced a markedly different response profile. There was no measurable increase in Zn concentrations in the leaves under either seed nanotreatment. In contrast, Zn concentrations increased in the root, and 8 nm ZnO nanopriming resulted in higher root Zn content compared with 45 nm ZnO seed treatment. Cu displayed an organ-specific shift, increasing slightly in the shoot but decreasing in the root as a result of ZnO nanopriming. Due to ZnO nanopriming, the concentrations of Mn, Fe, and Co consistently decreased in both tissues, exhibiting a pattern opposite to that observed under Zn deficiency. Overall, Fe, Mn, and Co showed tightly coordinated behavior: their concentrations increased under Zn deficiency but declined following nZnO priming. Although nZnO treatment enhanced Zn content in the root, the total Zn content of the plants remained far below that of Zn-sufficient plants. The nanoparticles likely released Zn that contributed to the moderate increase *in planta* Zn content and supported biomass improvement; however, this cannot fully explain the positive effects of nZnO. This suggests that additional mechanisms, beyond Zn ion supply, contribute to the growth-promoting impact of nZnO seed priming (Table 1).

**Table 1:** Concentration of Microelements (μg/g Dry Weight, DW; Zn, B, Mn, Fe, Ni, Co, Cu) in the Leaves and Roots of Three-Week-Old Tomato Plants Grown from Primed Seeds (Hydroprimed, HP; 100 mg·L^–1^ 8 nm nZnO; 100 mg·L^–1^ 45 nm nZnO) and Cultivated with Optimal (Zn+) or Suboptimal (Zn−) Zn Supply^a^

		Element concentrations inside the plant part [ppm (μg/g DW)] with ± SD (SD: (RSD × Mean)/100)						
Sample type	Treatment	Zn	B	Mn	Fe	Ni	Co	Cu
Leaf	HP Zn+	18.6 ± 0.4	595 ± 0.8	104.2 ± 1.0	100.2 ± 3.6	<0.2	1.0 ± 0.0	28.5 ± 0.3
	8 nm nZnO Zn+	22.0 ± 0.8	613 ± 0.8	125.2 ± 1.9	117.0 ± 1.2	<0.2	0.6 ± 0.0	32.9 ± 0.5
	45 nm nZnO Zn+	37.0 ± 0.6	570 ± 2.3	120.7 ± 1.3	114.9 ± 2.4	<0.2	0.4 ± 0.0	31.4 ± 0.3
	HP Zn–	<0.2	517 ± 1.8	126.7 ± 2.8	345.4 ± 4.0	<0.2	0.7 ± 0.0	15.0 ± 0.4
	8 nm nZnO Zn–	<0.2	416 ± 0.8	74.8 ± 2.1	222.5 ± 0.9	<0.2	1.4 ± 0.1	13.6 ± 0.4
	45 nm nZnO Zn–	<0.2	449 ± 1.9	73.4 ± 0.8	204.2 ± 2.6	<0.2	1.0 ± 0.0	17.2 ± 0.1
Root	HP Zn+	1176.3 ± 30.5	283 ± 2.0	797.1 ± 10.2	947.5 ± 4.9	<0.2	38.1 ± 1.0	448.7 ± 10.9
	8 nm nZnO Zn+	1135.7 ± 35.2	293 ± 1.0	709.2 ± 5.6	1230.1 ± 9.8	2.6 ± 0.3	40.8 ± 1.0	376.3 ± 14.1
	45 nm nZnO Zn+	965.5 ± 31.8	265 ± 0.4	688.2 ± 12.7	1023 ± 8.0	1.5 ± 0.1	36.8 ± 0.8	423.9 ± 8.2
	HP Zn–	<0.2	263 ± 0.9	3213.5 ± 529	1520.8 ± 29.1	5.0 ± 1.0	68.3 ± 2.4	338.8 ± 17.5
	8 nm nZnO Zn–	37.4 ± 0.9	247.3 ± 3.6	1982.1 ± 33.5	1031.2 ± 26.9	2.5 ± 0.3	41.8 ± 0.8	260.4 ± 8.4
	45 nm nZnO Zn–	9.7 ± 0.2	233 ± 0.5	2488.7 ± 38.8	925.1 ± 14.1	1.9 ± 0.3	43.9 ± 0.7	246.8 ± 6.3

a Data are presented as mean ± SD, n = 3.

### 3.7 Reactive Oxygen/Nitrogen Species Levels and Antioxidant Enzyme Responses

Beyond whole-organ quantification of Zn, Zn levels were detected at the tissue level in the primary root tips of tomato plants, which are ideal for fluorescent microscopic visualization (Figure 5A). In healthy plants, Zn levels were increased by seed priming with both nanoparticle sizes , and the larger nanoparticles caused significantly higher Zn levels compared to hydroprimed plants. Suboptimal Zn supply resulted in decreased Zn levels in the root tip (∼60% decrease) of hydroprimed plants, while seed priming with either 8 nm or 45 nm nZnOs significantly increased Zn levels (by ∼20%) in the primary root tip of Zn-deficient tomato plants, but levels remained notably lower than in plants grown with full Zn (Figure 5A).

**Figure 5:**
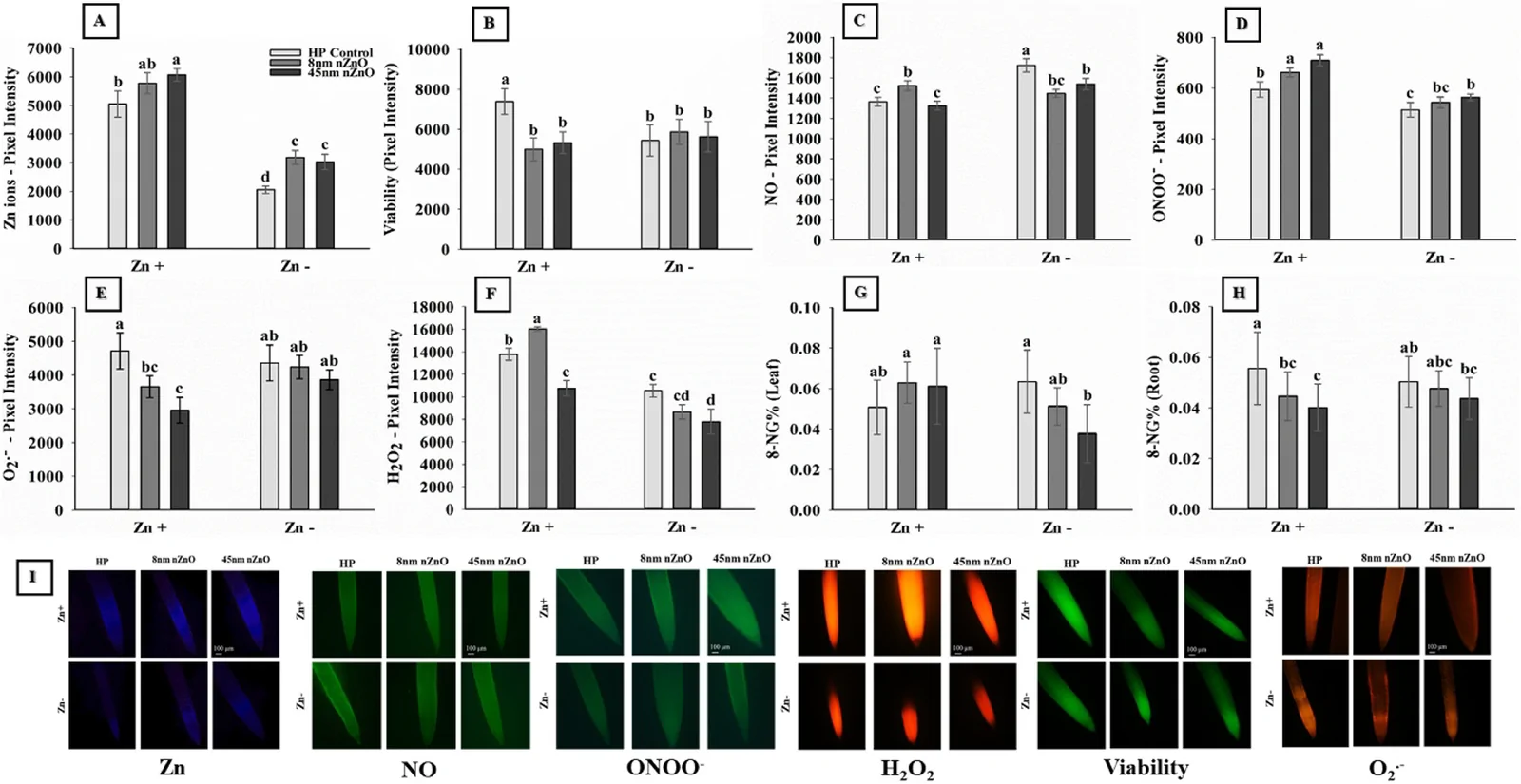
Levels of intracellular Zn ions (A), cell viability (B), nitric oxide (NO, C), peroxynitrite (ONOO^–^, D), superoxide anion radical (•O_2_^–^, E), hydrogen peroxide (H_2_O_2_, F) in the primary root tips of vegetative-stage tomato grown with optimal (Zn +) or suboptimal (Zn−) Zn supply. Tomato seeds were primed with distilled water (HP control), 8 nm nZnO (100 mg·L^–1^) or 45 nm nZnO (100 mg·L^–1^). Level of 8-nitroguanine (8-NG, % of the total RNA) in the leaves (G) and roots (H) of tomato. Different letters indicate statistically significant differences according to Duncan’s test (*p* < 0.05). (I) Representative microscopic images showing primary root tips of tomato plants treated and cultivated as mentioned above. Scale bars = 100 μm.

Interestingly, nZnO seed priming resulted in reduced cell viability of the primary root tip in plants grown with adequate Zn supply, and the extent of the decrease (∼30%) was similar to that caused by Zn deficiency. Plants grown from nZnO-primed seeds showed a similar reduction in root cell viability as hydroprimed plants (Figure 5B).

In plants grown with optimal Zn supply, both NO and ONOO^–^ levels were increased by ∼8 nm nZnO seed priming, while 45 nm nZnO increased only ONOO^–^ levels. In Zn-deficient, hydroprimed plants, the NO level significantly increased, while nZnO priming notably reduced it (Figure 5C). In contrast, Zn deficiency resulted in reduced ONOO^–^ levels, which were elevated by 45 nm nZnO seed priming (Figure 5D).

We suspected an *in planta* antioxidant-inducing nanozyme function of nZnO; therefore, we evaluated the levels of reactive molecules and antioxidant enzymes in seed-primed tomato plants. Regarding reactive oxygen species, nZnO (8 nm) seed priming caused decreased •O_2_^–^ levels and increased H_2_O_2_ levels in the root tips, while larger nanoparticles significantly decreased the level of both molecules in plants adequately supplied with Zn (Figure 5E,F). Under Zn deficiency, ZnO nanopriming exerted no effect on •O_2_^–^ levels but further decreased H_2_O_2_ levels (Figure 5E,F).

As a consequence of the accumulation of reactive nitrogen species, the formation of 8-nitroguanine (8-NG) as an indicator of RNA nitration was quantified in the leaves and roots of tomato (Figure 5G,H). In the case of full Zn supply, nZnO priming caused no significant alteration in the 8-NG level in the leaf, but led to a significant decrease in the root system. Moreover, suboptimal Zn supply slightly (not significantly compared to the hydroprimed control) enhanced the 8-NG level in the leaf, which was significantly decreased by seed priming with ∼45 nm nZnO (Figure 5G). In the root system of optimally Zn-supplied tomato plants, 45 nm nZnO seed priming led to the significant diminution of the 8-NG level, while neither Zn deficiency itself nor nZnO seed priming in Zn-deficient roots caused any significant changes in 8-NG levels (Figure 5H).

SOD activity was not significantly modified by nZnO seed priming in plants cultivated with optimal Zn supply (Figure 6A,B). Zn-deficient conditions resulted in activation of leaf SOD, which was significantly reduced by seed nZnO priming. In the root system, 45 nm nZnO increased SOD activity in Zn-deficient plants. In the leaves of hydroprimed plants, CAT activity was significantly diminished by suboptimal Zn supply (Figure 6C,D), while nZnO seed priming significantly increased CAT activity. Interestingly, root CAT did not respond to either Zn deficiency or nZnO seed priming. In both organs of hydroprimed plants, Zn deficiency significantly activated APX activity (Figure 6E,F), while nZnO seed priming caused reduced activity. Regarding *in planta* POD (Figure 6G,H), inadequate Zn levels caused enhanced activity in the leaves and decreased activity in the roots of hydroprimed plants. nZnO seed priming did not significantly modify POD activity either in the roots or in the leaves. *In vitro* peroxidase-like activity of nZnOs was evaluated using TMB as a chromogenic substrate.^33^ In the presence of H_2_O_2_, all nZnOs catalyzed the oxidation of TMB, resulting in the formation of a purple-blue product with a maximum absorption peak at ∼560 nm (Figure 6I). The control reaction without nZnOs showed negligible color change and no noticeable absorption peaks.

**Figure 6:**
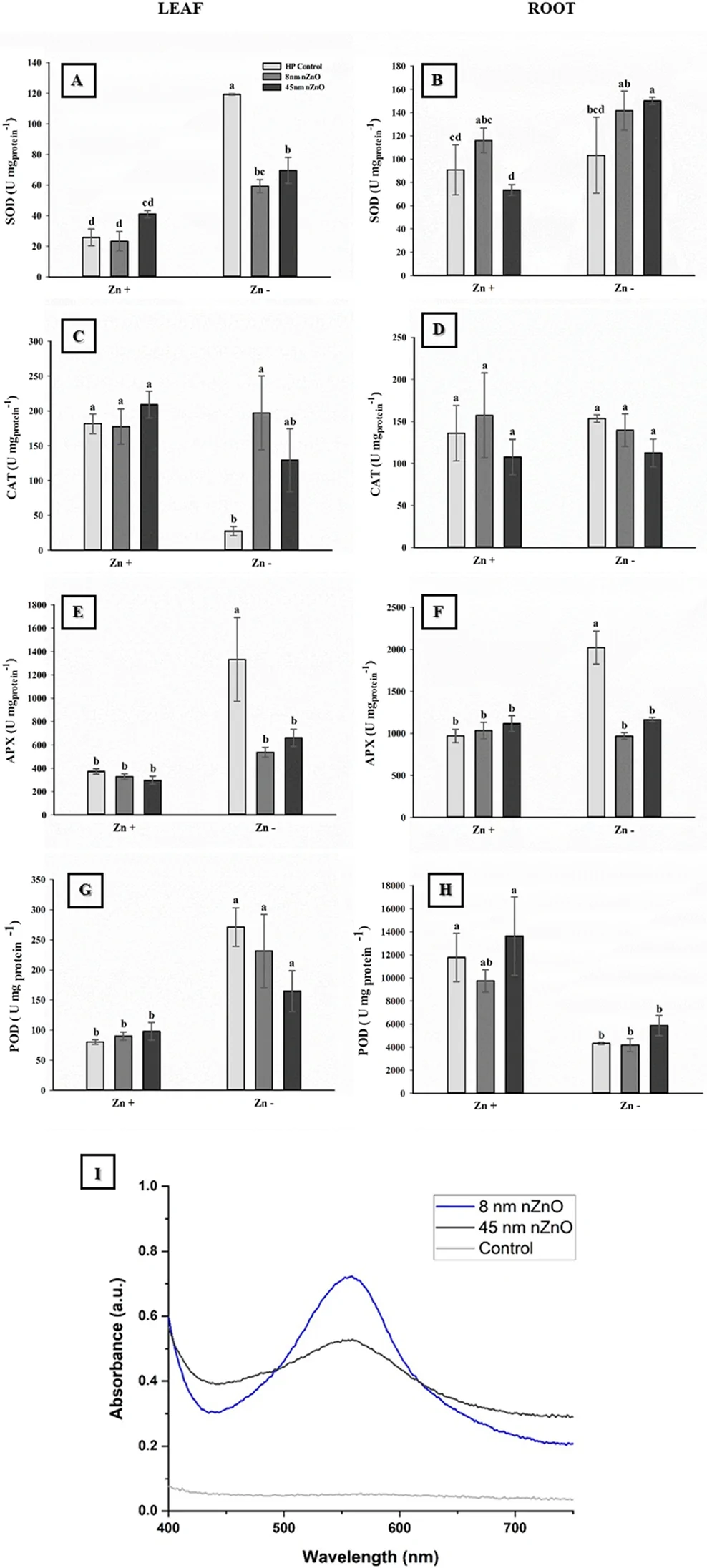
Activity of superoxide dismutase (SOD; A, B), catalase (CAT; C, D), ascorbate peroxidase (APX; E, F) and cell wall peroxidases (POD; G, H) in the leaf (A, C, E, G) and root (B, D, F, H) of three-week-old tomato grown from seeds primed with distilled water (HP), 8 nm nZnO (100 mg·L^–1^) or 45 nm nZnO (100 mg·L^–1^) and cultivated with optimal (Zn +) or suboptimal (Zn−) Zn supply. Different letters indicate statistically significant differences according to Duncan’s test (*p* < 0.05). POD-like activity of nZnOs (I). UV–vis absorption spectra of TMB oxidation in the presence of H_2_O_2_ without nZnOs (control) and with small (∼8 nm) or large (∼45 nm) nZnOs (I).

### 3.8 Integrated Metabolic Reprogramming in Leaves and Roots under nZnO Seed Priming

To explore the broader effects of nanopriming, we examined changes in metabolites—including amino acids, organic acids, sugars and polyamines—in the leaves and roots of Zn-sufficient and Zn-deficient tomato plants following nZnO seed priming. Under Zn-sufficient conditions, nZnO priming decreased succinic acid and increased fumaric acid concentrations in the leaves, accompanied by reduced arabinose, cellobiose, and trehalose contents and an increase in raffinose. In contrast, under Zn-deficient conditions, nZnO priming, compared with hydropriming, decreased the levels of l-serine, l-lysine, tryptophan, fumaric acid, and 2-ketoglutaric acid, while increasing succinic and malic acid contents in the leaves, and 45 nm nZnO showed slightly larger effects than 8 nm nZnO (Figure 7; Table S1). Regarding leaf carbohydrates, nZnO priming caused a significant decrease in arabinose, galactose, cellobiose, and trehalose concentrations. In the roots of Zn-sufficient plants, nZnO seed priming led to reduced valine, serine, and putrescine levels, together with decreased galactose and increased mannose contents. Conversely, in the roots of Zn-deficient plants, nZnO priming increased the levels of l-serine, l-lysine, 1,3-diaminopropane, arabinose, and cellobiose, while reducing D-fructose, d-glucose, and D-mannose contents, and 45 nm nZnO showed slightly more pronounced effects than 8 nm nZnO (Figure 7; Table S1, 2).

**Figure 7:**
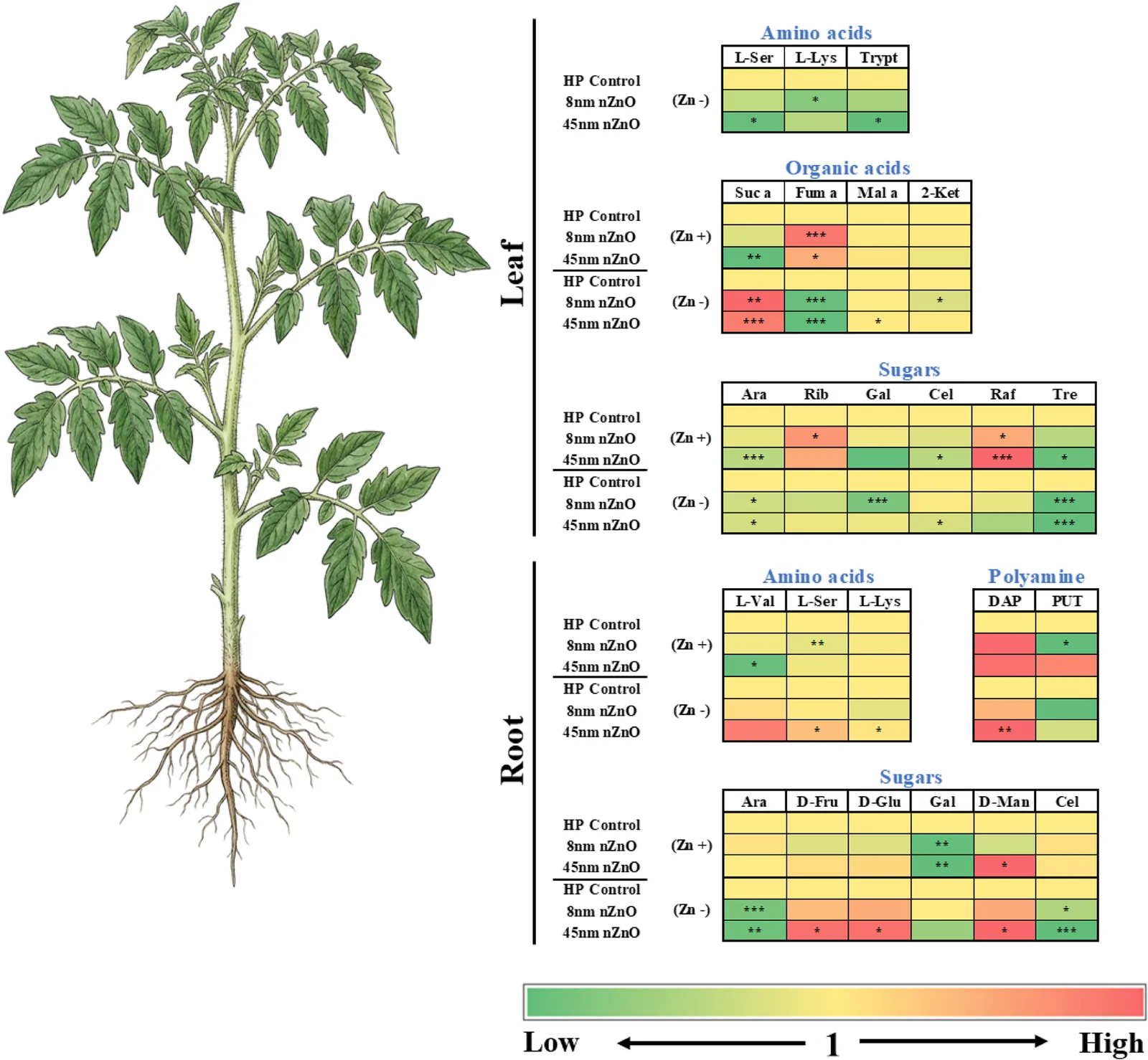
Schematic representation of the results of metabolite analysis in the leaves and roots of tomato grown from seeds primed with distilled water (HP), 8-nm nZnO (100 mg·L^–1^) or 45-nm nZnO (100 mg·L^–1^) and cultivated with optimal (Zn +) or suboptimal (Zn−) Zn supply. The heat map represents the fold change of each value compared with the corresponding untreated control, and color intensity reflects relative changes compared with the untreated control (control = 1). l-Ser= l-serine, l-Lys = l-lysine, Trypt = tryptophan, Suc a = succinic acid, Fum a = fumaric acid, Mal a = malic acid, 2-Ket = 2-ketoglutaric acid, Ara = arabinose, Rib = ribose, Gal = galactose, Cel = cellobiose, Raf = raffinose, Tre = trehalose, l-Val = l-valine, DAP = 1,3-diaminopropane, PUT = putrescine, D-Fru = D-fructose, d-Glu = d-glucose, D-Man = D-mannose. Asterisks indicate that values were significantly different from those of hydroprimed control plants grown with optimal Zn supply or hydroprimed control plants with suboptimal Zn supply at *p* < 0.05 according to *a t* test. All data were extracted from Tables S1 and S2.

### 3.9 Transcriptomic Analysis

To elucidate the molecular responses underlying nZnO-mediated alleviation of Zn deficiency-induced growth inhibition, transcriptomic profiling was performed in leaves and roots by comparing hydroprimed and nZnO-primed plants under different Zn supply conditions (Tables S3 –S6). Principal component analysis revealed clear separation of treatment groups in leaf tissues, whereas root tissues showed more overlap among transcriptomic profiles, indicating tissue-specific responses to Zn deficiency and nZnO priming (Figure S5). Since seed priming with 45-nm nZnO improved biomass production in both shoots and roots, this nanoparticle size was selected for downstream analyses.

In total, 2,611 DEGs were identified in the leaves of hydroprimed Zn-deficient tomato plants, of which 361 were upregulated (13.8%) and 2250 were downregulated (86.1%) (Table S3). Furthermore, 31 upregulated (10.3%) and 268 downregulated (89.6%) genes were identified in the leaves of nZnO-primed Zn-deficient tomato plants compared to hydroprimed Zn-deficient tomato (Table S5). In total, 205 DEGs were identified in the roots of hydroprimed Zn-deficient tomato plants, of which 55 were upregulated (26.8%) and 150 were downregulated (73.1%) compared to plants grown with full Zn (Table S4). 112 upregulated (97.3%) and 3 downregulated (2.6%) genes were identified in the roots of nZnO-primed Zn-deficient tomato plants compared to roots of plants grown from hydroprimed seeds under Zn-deficient conditions (Table S6).

Venn analysis revealed a subset of genes (12) commonly regulated under Zn deficiency and nZnO priming. In leaves, shared upregulated genes are associated with photosynthesis, chlorophyll biosynthesis, and protein turnover, consistent with partial maintenance of photosynthetic capacity under Zn deficiency (Figure 8A). This was supported by increased chlorophyll content following nZnO priming (Figure S6). In contrast, a large set of stress- and defense-related genes, including those associated with ROS detoxification, pathogen response, and phytohormone signaling, remained downregulated under both conditions (Figure 8B), suggesting that nZnO priming does not broadly activate stress-responsive transcriptional programs in leaves. There were no overlapping downregulated genes between Zn-deficient roots and nZnO-treated Zn-deficient roots (Figure 8D), and only one gene (ethylene response factor 2) was upregulated by both conditions in the root (Figure 8C).

**Figure 8:**
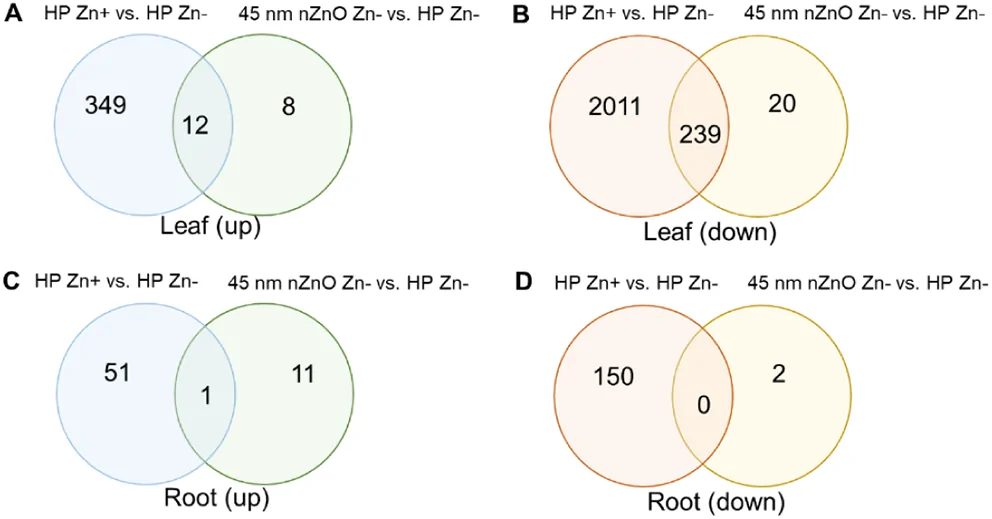
Venn diagram analysis showing the overlap of upregulated (up) or downregulated (down) genes among comparisons in leaves (A, B) and roots (C, D) of tomato grown from seeds primed with distilled water (HP), 8-nm nZnO (100 mg·L^–1^) or 45-nm nZnO (100 mg·L^–1^) and cultivated with optimal (Zn +) or suboptimal (Zn−) Zn supply.

To identify the main pathways through which nZnO contributed to enhanced Zn deficiency tolerance, the top 10 KEGG pathways were analyzed in tomato leaves and roots of plants primed either with water or 45 nm nZnO, grown with suboptimal or optimal Zn supply (Figure 9). KEGG enrichment analysis highlighted photosynthesis- and redox-related pathways as dominant in Zn-deficient leaves, while nZnO priming was mainly associated with glutathione metabolism and carbohydrate-related pathways (Figure 9A,B). In roots, Zn deficiency affected sulfur- and amino acid-related metabolism, whereas nZnO priming preferentially enriched phenylpropanoid- and amino acid metabolism-related pathways (Figure 9C,D). Overall, these results suggest that nZnO priming modulates a limited number of core metabolic hubs rather than inducing broad-scale pathway reprogramming.

**Figure 9:**
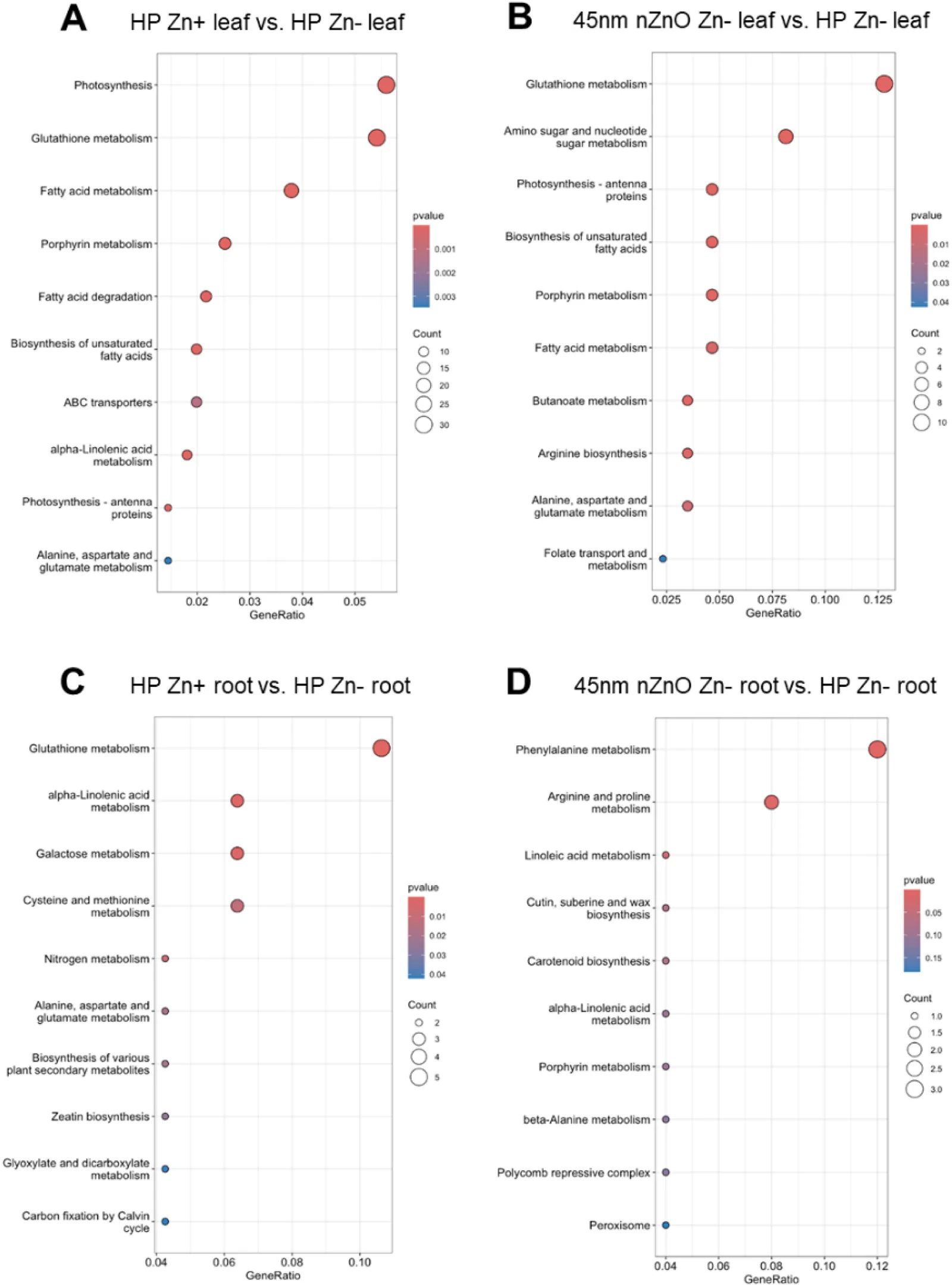
KEGG pathway enrichment analysis of the differentially expressed genes among the comparisons in leaves (A, B) and roots (C, D) of tomato grown from seeds primed with distilled water (HP), 8-nm nZnO (100 mg·L^–1^) or 45-nm nZnO (100 mg·L^–1^) and cultivated with optimal (Zn +) or suboptimal (Zn−) Zn supply. Count = number of DEGs.

At the gene level, Zn deficiency strongly affected multiple functional categories in leaves, including antioxidant defense, phytohormone signaling, and primary metabolism. This included downregulation of representative genes such as glutathione *S*-transferases (GSTs), catalases, and peroxidases, as well as genes associated with auxin and ethylene signaling. nZnO priming partially attenuated these transcriptional changes (Figure 10A; Tables S3, S5), suggesting a moderation of Zn deficiency-associated stress responses at the transcript level. In roots, transcriptional changes were less pronounced and affected a more limited set of genes. nZnO priming mainly influenced genes associated with phenylpropanoid metabolism, amino acid metabolism, and ethylene biosynthesis, including PAL-like genes, polyamine oxidases, and ACC oxidase-related transcripts (Figure 10B; Tables S4, S6).

**Figure 10:**
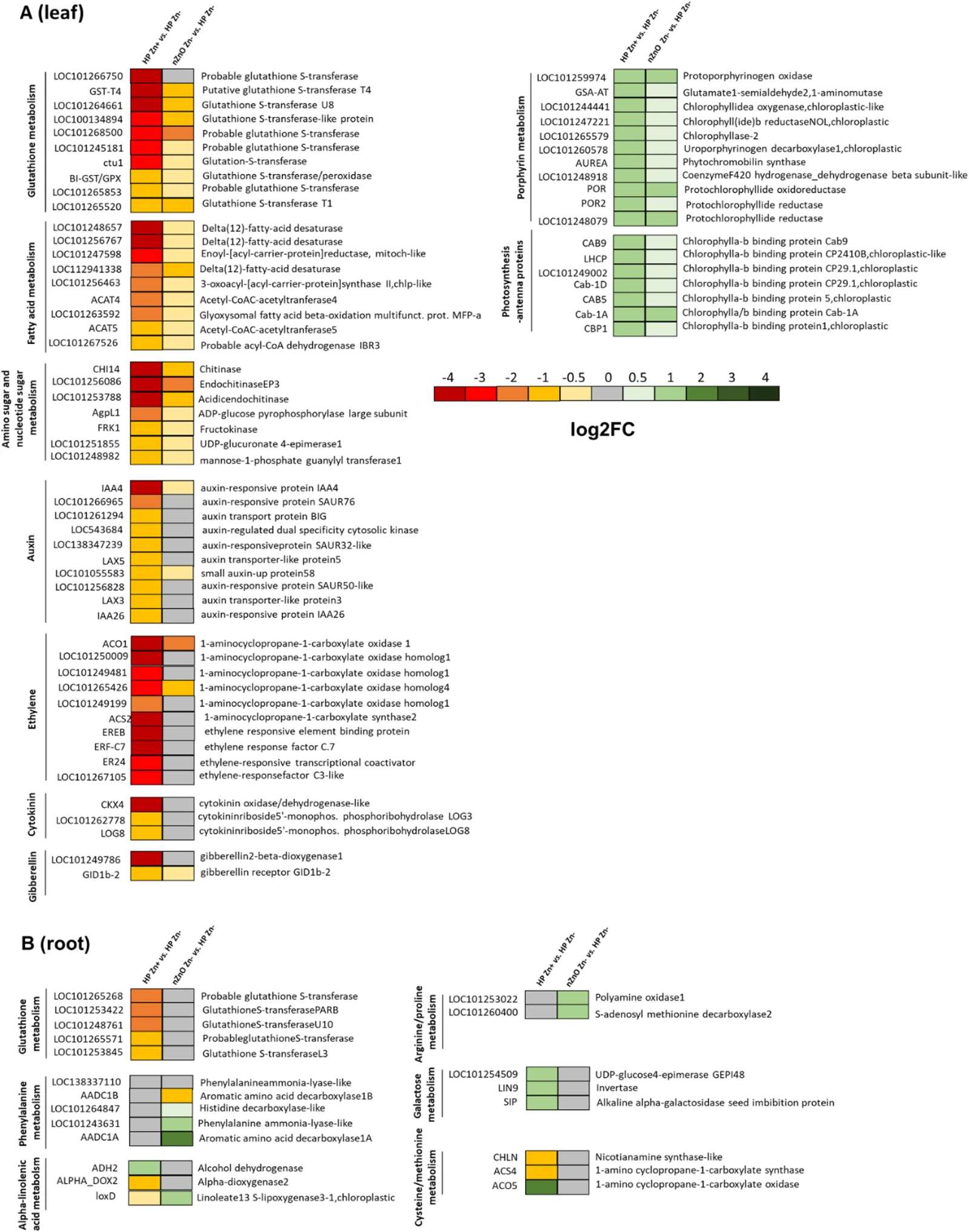
Selected differentially expressed genes involved in glutathione metabolism; fatty acid metabolism; amino sugar and nucleotide sugar metabolism; porphyrin metabolism; photosynthesis-antenna proteins; phenylalanine metabolism; α-linolenic acid metabolism; arginine and proline metabolism; galactose metabolism; and cysteine and methionine metabolism, as well as auxin, ethylene, cytokinin, and gibberellin metabolism and signaling in leaves (A) and roots (B) of tomato plants grown from seeds primed with distilled water (HP), 8-nm nZnO (100 mg·L^–1^), or 45-nm nZnO (100 mg·L^–1^) and cultivated under optimal (Zn+) or suboptimal (Zn−) Zn supply. The heat map indicates log_2_ fold change (FC) in gene expression.

## 4 Discussion

In this study, the effect of nZnO seed priming is monitored from the seed through the seedling stage to Zn-deficiency-modified vegetative growth.

The TEM images indicated the uptake of both the 8 and 45 nm nZnOs by tomato seeds, reaching the embryo (Figure 1). While smaller particles formed aggregates within the plant tissues, larger-sized nZnOs seem to enter cells without undergoing structural transformation. Moreover, nZnOs of different sizes are differentially compartmentalized within the tomato embryo: aggregates of small nZnOs are localized in vesicle-like structures, while larger nZnOs are present in lipid bodies of the embryo cotyledon. Although lipid bodies are more commonly associated with protein-rich legume seeds^52^ or oilseeds such as olive,^53^ the characteristic toluidine blue staining pattern and the spherical morphology of these vesicles support the presence of lipid bodies in tomato seeds (Figure 1). Inside the cells, nanoparticles may transform and create lipid-nanoparticle complexes that may localize inside or around lipid bodies; however, the biological relevance of this needs further evaluation. Based on the seed surface analysis, the 8 nm nZnOs reduce hairiness, which may weaken the protection of the seed/embryo, while hydroprimed control and 45 nm nZnOs are biocompatible and do not cause detectable damage to seed coat trichomes (Figure S2). It is also conceivable that the presence of trichomes may assist the adhesion of the nanoparticles to the seed surface; however, their internalization through trichomes has not been evidenced.^54^ μXRF analysis revealed that nZnO priming is a more effective strategy than bulk Zn priming for enriching tomato seeds with Zn (Figure 2). However, this enrichment was predominantly localized in the outer seed layers, limiting direct Zn supply to the embryo. A plausible explanation is that nZnO and bulk Zn induce ROS at the seed surface, oxidizing polyphenols and flavonoids that form complexes with Zn and promote its retention in the seed coat.^55^ Consistent with this mechanism, the degree of seed coat browning (Figure S1) may be associated with Zn levels detected on the seed surface (Figure 2). Excess Zn delivered by 45 nm nZnO or bulk Zn perturbed seed nutrient homeostasis, possibly by competitively inhibiting Ca, Fe, and Cu binding and transport, displacing these ions from metal–ligand complexes and altering metal transporter activity.^56^ In contrast, dissolution measurements performed under the seed-priming conditions showed that the ∼8 nm nZnO released a substantially greater proportion of dissolved Zn^2+^ than the ∼45 nm particles. This greater Zn^2+^ release is consistent with the higher specific surface area and likely contributes to their more efficient Zn accumulation, enabling controlled Zn incorporation without triggering broad cation competition or redistribution in the seed. In tomato seedlings germinated from primed seeds, Zn was poorly detectable by LIBS and, in contrast to seeds, 8 nm nZnO did not, but 45 nm nZnO did, cause significant elevation of the Zn signal, due to the fact that smaller nanoparticles may be more readily dissolved and assimilated into metabolic pools (e.g., incorporated into enzymes, proteins, transcription factors), which is not detectable by LIBS (Figure 3). Overall, this suggests that nanoparticle size strongly influences Zn persistence, detectability, and possibly its transport and utilization in tomato seedlings. Sodium exhibited better detectability and more pronounced treatment-dependent changes than Zn, indicating that Na levels are highly sensitive to nanoparticle-induced physiological alterations. It is well documented that nZnO improves salt tolerance by decreasing *in planta* Na concentration.^57^ In this experimental system, elevated Na levels observed in nonstressed seedlings after 8 nm nZnO priming suggest that localized Zn release and redox imbalance may interfere with Na transporters or channels, thereby reducing ionic selectivity and promoting Na accumulation in both roots and hypocotyls. The pronounced increase in the Ca signal in the roots of seedlings derived from 8-nm nZnO-primed seeds suggests activation of Ca-mediated stress signaling pathways, likely in response to metal-induced stress.^58^ In contrast, Ca accumulation following 45-nm nZnO or bulk Zn priming was more localized near the root-shoot junction, indicating a weaker or spatially confined stress response. Changes in Mg distribution, characterized by increased hypocotyl levels and reduced root levels (particularly following 45-nm nZnO and bulk Zn priming), suggest a redistribution of Mg toward actively growing shoot tissues, possibly reflecting altered metabolic demands under excess Zn availability. Overall, these results indicate that smaller ZnO nanoparticles predominantly elicit stress-related ionic responses, whereas larger nanoparticles provide a more controlled Zn source, underscoring particle size as a key determinant of Zn bioavailability and nutrient homeostasis during early seedling development.

In vegetative-stage tomatoes, the shoot system was more sensitive to Zn deficiency than the roots, as has also e.g. in maize.^59^ However, root fresh weight was significantly reduced by suboptimal Zn supply, potentially due to a stomatal closure-triggered decrease in water uptake.^60,61^ Overall, 45 nm nZnO exerted a positive effect on biomass production, while 8 nm nZnO showed a root-specific growth-promoting effect under Zn-deficient conditions. Notably, nZnO seed priming improved the growth of Zn-deficient plants to levels comparable with those of Zn-sufficient plants and exhibited broader and more pronounced promoting effects than bulk Zn priming (Figure 4). This may be primarily due to the fact that ZnO has a larger surface area-to-volume ratio than bulk Zn compounds, which allows more controlled and rapid dissolution into readily utilized Zn ions.

Inadequate Zn supply caused a reduction in the amount of Zn ions, which was accompanied by the accumulation of Fe, Mn, and Co mainly in the root system of hydroprimed plants (Table 1). The antagonism between Zn and the above microelements may be due to upregulation of broad-specificity metal transporters (*e*.*g*., ZIP, IRT1, NRAMP);^62^ however, RNA sequencing showed that most of the metal transporters are downregulated by suboptimal Zn supply in tomato roots (Table S4). Importantly, dissolution measurements carried out under seed-priming conditions demonstrated that 8 nm nZnO exhibited approximately 2-fold greater Zn^2+^ release than 45 nm nZnO after 24 h. Nevertheless, the ionomic profile, together with the limited increase in total Zn content, suggests that Zn ion release alone cannot fully explain the observed effects. Rather, the root-associated accumulation of microelements may reflect their limited root-to-shoot translocation. This is supported by the slight downregulation of transporters involved in long-distance element transport (e.g., HMA, and the YSL family, Table S4).^63^ Copper levels remain stable during Zn deficiency (Table 1) which reflects highly specific and tightly regulated Cu transport systems. However, three genes encoding Cu transporters (copper transporter ATX1, copper transporter 5, copper transporter 6-like) are downregulated by suboptimal Zn supply in the leaf (Table S3). More importantly, the alleviation of nonspecific metal overaccumulation by nZnO priming is likely a consequence of restored Zn availability. The decrease in *in planta* Zn levels associated with suboptimal Zn supply was clearly detectable at the tissue level. Seed priming with nZnO effectively supplied Zn for vegetative-stage tomato plants, leading to increased Zn levels in the lateral root tips (Figure 5A, I). Nevertheless, nZnO priming could not restore the reduced viability caused by Zn deficiency (Figure 5B,I), which suggests that Zn is present (indicated by an increased Zinquin signal), but not necessarily functionally integrated.

Available knowledge is limited regarding reactive nitrogen species-associated responses in Zn-deficient plants,^64^ especially in experimental systems involving seed priming. This motivated us to examine changes in NO and ONOO^–^ levels and the extent of nucleic acid nitration (Figure 5). In hydroprimed plants, suboptimal Zn supply may activate ONOO^–^ detoxification (e.g., peroxiredoxins, thioredoxins) and trigger NO production, while nZnO priming reverses these trends. Zn deficiency induced elevated NO levels in tomato roots, similar to those in other moderately or highly sensitive species like *Arabidopsis thaliana*, *Brassica napus*, and *Zea mays*.^64,65^ A possible mechanism of Zn deficiency-induced NO production may be the accumulation of iron (Table 1) which in turn may induce NO production.^66,67^ In the roots of optimally Zn-supplied plants, nZnO increased ONOO^–^ levels and simultaneously attenuated RNA nitration (8-NG levels), which suggests selective degradation of nitrated nucleic acids. Zn deficiency did not affect RNA nitration compared to optimal Zn supply. The 45 nm nZnO seed-priming-triggered reduction in 8-NG levels in the leaves of Zn-deficient tomato reflects the possible activation of degradation mechanisms with the aim of detoxification. However, specific proteins that regulate 8-NG levels have not yet been identified in plants.^68^

Antioxidant responses revealed a clear reprogramming of defense pathways (Figure 6). Zn deficiency activated the leaf SOD-APX-POD pathway, effectively detoxifying superoxide-derived H_2_O_2_, while CAT activity was downregulated. These Zn deficiency-induced changes in protein activities may be associated with changes in gene expression. For instance, suboptimal Zn supply resulted in upregulation of genes encoding Cu/Zn SOD, the copper chaperone for SOD, chloroplastic Cu/Zn SOD, and L-ascorbate peroxidase, and in strong downregulation of catalase in tomato leaves (Table S3). In contrast, nZnO priming strongly activates CAT without upregulating the corresponding genes, while downregulating the SOD-APX-POD pathway, shifting H_2_O_2_ detoxification toward a CAT-dominated system. nZnO-associated downregulation of genes encoding SOD1 and several peroxidases supports the molecular-level regulation of the antioxidant system (Table S5). The reprogramming of enzymatic antioxidants likely represents an adaptive strategy, as CAT provides an energetically efficient mechanism for bulk H_2_O_2_ removal while preserving ascorbate for other essential cellular functions. This is supported by the results of other studies where nZnO increased the activity of catalase in plant tissues.^69,70^ ZnO nanoparticles of both sizes exhibited peroxidase-like activity in cell-free assays (Figure 6I), indicating intrinsic nanozyme potential.^3,3^ In contrast, no corresponding increase in endogenous POD activity was observed in either Zn-supplied or Zn-deficient tomato plants following nZnO seed priming (Figure 6G,H). This discrepancy highlights that nanozyme activity measured in cell-free systems does not necessarily predict biological responses *in planta*. Whereas ZnO nanoparticles directly catalyze peroxidase-like reactions in a cell-free environment, endogenous POD activity is determined by the abundance, isoform composition, and transcriptional and post-translational regulation of plant heme peroxidases within the cellular redox network. Therefore, the observed *in vitro* nanozyme activity reflects the intrinsic catalytic properties of nZnO but should not be considered a direct predictor of its biological effects *in planta*.

Primary metabolism has been found to be modulated by metal nanoparticles, including nanocerium,^71^ nanocopper^72^ and nZnO in iron-deficient plants.^73^ Here, we first observed that nZnO seed priming induced distinct, organ-specific metabolic adjustments that were strongly dependent on Zn nutritional status (Figure 7; Table S1, S2). Under Zn-sufficient conditions, nZnO priming was associated with a coordinated metabolic profile in leaves characterized by reduced succinate, elevated fumarate, and increased raffinose accumulation, consistent with enhanced tricarboxylic acid (TCA) cycle activity and a primed physiological state rather than the induction of stress metabolism. In contrast, under Zn-deficient conditions, nZnO priming disrupted TCA cycle continuity, as evidenced by succinate and malate accumulation with concomitant depletion of fumarate and 2-oxoglutarate, and suppressed amino acid biosynthesis, suggesting that Zn limitation may override the beneficial metabolic adjustments associated with priming under Zn sufficiency. Regarding leaf carbohydrates, the pronounced reduction in arabinose, galactose, cellobiose, and trehalose following nZnO priming is consistent with altered cell wall carbohydrate metabolism and trehalose-related signaling,^74^ suggesting modified carbon allocation following nZnO priming. nZnO seed priming differentially affected root metabolism depending on Zn availability, being associated with restrained nitrogen and polyamine metabolism and altered carbohydrate homeostasis under Zn sufficiency, whereas under Zn deficiency it was associated with enhanced amino acid and polyamine metabolism and changes in carbohydrate composition consistent with a shift from soluble sugars toward structural carbohydrates (Figure 7; Tables S1, S2).

Zn deficiency caused strong transcriptional repression, particularly in tomato leaves (Figures 8–10; Table S3) similar to the aboveground tissues of wheat, where, e.g., GST genes, chitinase genes, and several genes involved in phytohormone signaling were downregulated.^75^ nZnO seed priming partially restored the Zn-deficiency transcriptome. For instance, several GST genes were downregulated in the leaves and, to a lesser extent, in the roots by suboptimal Zn supply (Figure 10; Tables S3, S4) possibly due to the prioritization of essential metabolism over detoxification.^76^ nZnO seed priming partially alleviated the Zn-deficiency-induced downregulation of GST genes (Figure 10; Table S5). This response may reflect improved Zn availability and the maintenance of redox-related regulatory processes, although these mechanisms were not directly examined in the present study. Also, several auxin-associated genes were downregulated in Zn-deficient leaves (Figure 10; Table S3), which may contribute to inadequate Zn availability-associated limitation of auxin synthesis, as was observed in diverse plant species.^77^ Moreover, a few cytokinin- and gibberellin-associated leaf genes were downregulated by suboptimal Zn supply (Figure 10; Table S3) suggesting the involvement of these hormonal pathways in the Zn-deficiency response of tomato; however, the available supporting evidence remains scarce.^77^ The downregulation of these phytohormone-linked genes may be associated with the decline in growth and development observed in Zn-deficient plants. The partial reversal of this expression pattern may contribute to the observed biomass improvement following nZnO seed priming. Similarly, some genes involved in fatty acid metabolism (e.g., desaturases, which are Zn-containing enzymes)^78^ showed Zn-deficiency-triggered downregulation, and nZnO seed priming reduced the rate of downregulation of these genes (Figure 10; Tables S3, S5) which may be associated with improved membrane function, although this was not directly assessed. Interestingly, ethylene-associated genes were regulated by Zn deficiency in an organ-dependent way, and in the roots, suboptimal Zn supply triggered activation of ACO (Figure 10; Tables S3, S4) suggests the role of ethylene as a stress hormone in the Zn deficiency response, similar to wheat.^75^ Restored Zn via nZnO seed priming eliminated this upregulation, reflecting that nZnO seed priming may mitigate Zn-deficiency-induced activation of ethylene biosynthesis-associated pathways. In the roots, nZnO seed priming resulted in the upregulation of a phenylalanine ammonia-lyase-like gene (LOC101243631) (Figure 10; Table S6) which is consistent with a potential activation of phenylpropanoid metabolism, the precursor pathway of flavonoids, lignans, and salicylic acid.^79^ Overall, nZnO seed priming resulted in relatively few DEGs in Zn-deficient tomato compared to hydropriming, suggesting fine-tuning rather than induction of a broad stress response.

## 5 Conclusions

Plants show multilevel responses to nanometal exposure, and the nanometal-associated effects on plant physiological processes are strongly influenced by both nanoparticle size and the plant’s developmental stage.^80^ Therefore, this study compared the effects of seed priming with smaller and larger nZnO at different stages of tomato development, from seeds to the Zn-deficient vegetative stage. During seed priming, both the 8 nm and 45 nm nZnOs were internalized into the seeds, reaching the embryo, and our results are the first to suggest that oil bodies act as intracellular storage sites for larger nZnOs. Using different methods (XRF, LIBS, ICP-MS, and fluorescence microscopy ), we established that the restoration of Zn availability by nZnO differs depending on the plant’s developmental stage: 8 nm nZnO has a better effect in seeds, while, in the long term, 45 nm nZnO provides better Zn availability in seedlings and vegetative plants. The growth inhibition caused by Zn deficiency was restored by 45 nm nZnO seed priming in both organs and had a significantly more favorable effect than bulk Zn. In addition to improving Zn availability, a CAT-associated *in planta* nanozyme effect also contributed to the beneficial effects of nZnO seed priming. To our knowledge, this is the first study to demonstrate that nZnO priming reduced RNA nitration in Zn-deficient tomato, which suggests a nitrosative stress-ameliorating effect in addition to the antioxidant effect. Under Zn deficiency, nZnO seed priming induces contrasting organ-specific metabolic reprogramming. Zn deficiency caused strong transcriptional repression, particularly in leaves, and nZnO seed priming partially restored the Zn-deficiency transcriptome.

These results support the conclusion that the nanopriming effect depends on nanoparticle size and may vary across plant developmental stages. Additionally, the nanopriming strategy applied here effectively improves plant fitness under Zn-deficient conditions through the combination of Zn supply restoration with antioxidant nanozyme effects. These observations indicate that nZnO seed priming may be a potentially useful strategy for improving tomato performance under Zn-deficient conditions. Nevertheless, its environmental impacts, scalability, and feasibility under field conditions for tomato production require further investigation.

## Supplementary Material











## Notes

Figures S1–S6: Seed morphology and priming effects; scanning electron microscopy (SEM) images of seed surfaces; normalized elemental contents in seed tissues; differences in Zn accumulation; principal component analysis (PCA) of elemental composition in seeds; total chlorophyll content of tomato plants under different Zn treatments Table S1–S2: Metabolite profiles, including amino acids, organic acids, sugars, and polyamines, in leaves and roots under different treatments (PDF)
Table S3: Differentially expressed gene classification in leaves (HP Zn+ vs HP Zn-) (XLSX)
Table S4: Differentially expressed gene classification in roots (HP Zn + vs HP Zn-) (XLSX)
Table S5: Differentially expressed gene classification in leaves (nZnO Zn- vs HP Zn-) (XLSX)
Table S6: Differentially expressed gene classification in roots (nZnO Zn- vs HP Zn-) (XLSX)

## References

[1] Sasmal D., Bhattacharya P. (2024). Understanding the vital role of Zn in plant nutrition. Int. J. Agric. Food Sci..

[2] Barrameda-Medina Y., Montesinos-Pereira D., Romero L., Blasco B., Ruiz J. M. (2014). Role of
GSH homeostasis under Zn toxicity in plants with different Zn tolerance. Plant Sci..

[3] Umair
Hassan M., Aamer M., Umer Chattha M. U., Haiying T., Shahzad B., Barbanti L., Nawaz M., Rasheed A., Afzal A., Liu Y., Guoqin H. (2020). The Critical
Role of Zinc in Plants Facing the Drought Stress. Agriculture.

[4] Sethi G., Behera K. K., Sayyed R., Adarsh V., Sipra B. S., Singh L., Alamro A. A., Behera M. (2025). Enhancing soil health
and crop productivity: the role of zinc-solubilizing bacteria in sustainable
agriculture. Plant Growth Regul..

[5] Sharma, V. ; Maurya, A. ; Kandhol, N. ; Singh, V. P. ; Sharma, S. ; Peralta-Videa, J. ; Tripathi, D. K. Zn deficiency in plants: an insight into fortification strategies. In Zn in Plants; Tripathi, D. K. ; Singh, V. P. ; Pandey, S. ; Sharma, S. ; Chauhan, D. K. , Eds.; Academic Press, 2025; pp. 249–264. DOI: 10.1016/B978-0-323-91314-0.00011-9.

[6] Zeng H., Wu H., Yan F., Yi K., Zhu Y. (2021). Molecular regulation
of Zn deficiency responses in plants. J. Plant
Physiol..

[7] Lv Z., Liu F., Zhang P., Li W., Song Q., Xu J. (2023). Revealing
the molecular mechanisms of Zn accumulation and Zn deficiency responses
in quinoa, analyzed by high-throughput gene expression profiling under
Zn depletion and resupply. Environ. Exp. Bot..

[8] Rani N., Chauhan A., Sagar N. A., Kumar V. (2025). Microbe-mediated regulation
in Zn-contaminated soils: the synergistic role of hyperaccumulator
plants and Zn-tolerant rhizobacteria. Front.
Agron..

[9] Çakmak İ., Kutman U. B. (2018). Agronomic biofortification
of cereals with Zn: a review. Eur. J. Soil Sci..

[10] Dimkpa C.
O., Bindraban P. S. (2018). Nanofertilizers:
new products for the industry?. J. Agric. Food
Chem..

[11] Semenova N. A., Burmistrov D. E., Shumeyko S. A., Gudkov S. V. (2024). Fertilizers
based
on nanoparticles as sources of macro-and microelements for plant crop
growth: A review. Agronomy.

[12] Kah M. (2015). Nanopesticides
and nanofertilizers: emerging contaminants or opportunities for risk
mitigation?. Front. Chem..

[13] Servin A., Elmer W., Mukherjee A., De la Torre-Roche R., Hamdi H., White J. C., Bindraban P., Dimkpa C. (2015). A review of the use of engineered nanomaterials to
suppress plant disease and enhance crop yield. J. Nanopart. Res..

[14] Ravishankar L. V., Puranik N., Lekkala V. V., Lomada D., Reddy M. C., Maurya A. K. (2025). ZnO Nanoparticles:
Advancing Agricultural Sustainability. Plants.

[15] Rajput V., Minkina T., Mazarji M., Shende S., Sushkova S., Mandzhieva S., Burachevskaya M., Chaplygin V., Singh A., Jatav H. (2020). Accumulation
of nanoparticles in
the soil-plant systems and their effects on human health. Ann. Agric. Sci..

[16] Lv Z., Sun H., Du W., Li R., Mao H., Kopittke P. M. (2021). Interaction
of different-sized ZnO nanoparticles with maize (Zea mays): Accumulation,
biotransformation and phytotoxicity. Sci. Total
Environ..

[17] Rastogi A., Zivcak M., Sytar O., Kalaji H. M., He X., Mbarki S., Brestic M. (2017). Impact of
metal and metal oxide nanoparticles
on plant: a critical review. Front. Chem..

[18] Mahakham W., Sarmah A. K., Maensiri S., Theerakulpisut P. (2017). Nanopriming
technology for enhancing germination and starch metabolism of aged
rice seeds using phytosynthesized silver nanoparticles. Sci. Rep..

[19] Acharya P., Jayaprakasha G. K., Crosby K. M., Jifon J. L., Patil B. S. (2020). Nanoparticle-mediated
seed priming improves germination, growth, yield, and quality of watermelons
(*Citrullus lanatus*) at multi-locations in Texas. Sci. Rep..

[20] Rico, C. Plant Exposure to Engineered Nanoparticles: uptake, Transformation, Molecular and Physiological Responses.; Academic Press, 2022.

[21] Mahra S., Tripathi S., Tiwari K., Sharma S., Mathew S., Kumar V., Sharma S. (2025). Harnessing
nanotechnology for sustainable
agriculture: from seed priming to encapsulation. Plant Nano Biol..

[22] Tripathi D. K., Shweta, Singh S., Singh S., Pandey R., Singh V. P., Sharma N. C., Prasad S. M., Dubey N. K., Chauhan D. K. (2017). An overview on manufactured
nanoparticles in plants: uptake, translocation, accumulation and phytotoxicity. Plant Physiol. Biochem..

[23] Dimkpa C., Adzawla W., Pandey R., Atakora W. K., Kouame A. K., Jemo M., Bindraban P. S. (2023). Fertilizers
for food and nutrition
security in sub-Saharan Africa: an overview of soil health implications. Front. Soil Sci..

[24] Molnár Á., Papp M., Zoltán Kovács D. Z., Bélteky P., Oláh D., Feigl G., Szőllősi R., Rázga Z., Ördög A., Erdei L., Rónavári A. (2020). Nitro-oxidative
signalling induced by chemically synthetized zinc oxide nanoparticles
(ZnO NPs) in Brassica species. Chemosphere.

[25] Molnár Á., Rónavári A., Bélteky P., Szőllősi R., Valyon E., Oláh D., Rázga Z., Ördög A., Kónya Z., Kolbert Z. (2020). ZnO nanoparticles induce cell wall
remodeling and modify
ROS/RNS signalling in roots of Brassica seedlings. Ecotoxicol. Environ. Saf..

[26] Bian S. W., Mudunkotuwa I. A., Rupasinghe T., Grassian V. H. (2011). Aggregation and
dissolution of 4 nm ZnO nanoparticles in aqueous environments: influence
of pH, ionic strength, size, and adsorption of humic acid. Langmuir.

[27] Srivastava V., Gusain D., Sharma Y. C. (2013). Synthesis,
characterization and application
of Zn oxide nanoparticles (n-ZnO). Ceram. Int..

[28] Tari I., Csiszar J., Horvath E., Poor P., Takács Z., Szepesi A. (2015). The alleviation of the adverse effects of salt stress
in the tomato plant by salicylic acid shows a time-and organ-specific
antioxidant response. Acta Biol. Cracov., Ser.
Bot..

[29] Dhindsa R. S., Plumb-Dhindsa P., Thorpe T. A. (1981). Leaf senescence: correlated with
increased levels of membrane permeability and lipid peroxidation,
and decreased levels of superoxide dismutase and catalase. J. Exp. Bot..

[30] Upadhyaya A., Sankhla D., Davis T. D., Sankhla N., Smith B. N. (1985). Effect
of paclobutrazol on the activities of some enzymes of activated oxygen
metabolism and lipid peroxidation in senescing soybean leaves. J. Plant Physiol..

[31] Bradford M. M. (1976). A rapid
and sensitive method for the quantitation of microgram quantities
of protein utilizing the principle of protein-dye binding. Anal. Biochem..

[32] Gallé Á., Farkas M., Pelsőczi A., Czékus Z., Kukri A., Dorner Z., Ördög A., Csiszár J., Bela K., Poór P. (2023). Contribution
of glutathione transferases in the selective and light-dependent effect
of flumioxazin on winter wheat (*Triticum aestivum* L.) and its typical weed common poppy (*Papaver rhoeas* L.). Agronomy.

[33] Liu P., Liang M., Liu Z., Long H., Cheng H., Su J., Tan Z., He X., Sun M., Li X., He S. (2024). Facile green synthesis of wasted hop-based zinc oxide nanozymes as
peroxidase-like catalysts for colorimetric analysis. Nanoscale.

[34] Kolbert Z. (2012). In vivo and
in vitro studies on fluorophore-specificity. Acta Biol. Szeged..

[35] Helmersson A., von Arnold S., Bozhkov P. V. (2008). The level of free intracellular Zn
mediates programmed cell death/cell survival decisions in plant embryos. Plant Physiol..

[36] Lehotai N., Kolbert Z., Pető A., Feigl G., Ördög A., Kumar D., Tari I., Erdei L. (2012). Selenite-induced hormonal
and signalling mechanisms during root growth of Arabidopsis thaliana
L. J. Exp. Bot..

[37] Yudiansyah Y., Ismiyanti I., Aziz S. A. (2024). Modification of the spectrophotometric
analysis protocol by sims d. and gamon to analyze leaf pigment content
using green spinach (*Amaranthus hybridus*) as a model
plant. J. Trop. Crop Sci..

[38] Sims D. A., Gamon J. A. (2002). Relationships between
leaf pigment content and spectral
reflectance across a wide range of species, leaf structures and developmental
stages. Remote Sens. Environ..

[39] Anders S., Pyl P. T., Huber W. (2015). HTSeq—a Python framework to
work with high-throughput sequencing data. Bioinformatics.

[40] Di
Tommaso P., Chatzou M., Floden E. W., Barja P. P., Palumbo E., Notredame C. (2017). Nextflow enables reproducible computational
workflows. Nat. Biotechnol..

[41] Ewels P. A., Peltzer A., Fillinger S., Patel H., Alneberg J., Wilm A., Garcia M. U., Di Tommaso P., Nahnsen S. (2020). The nf-core framework for community-curated
bioinformatics
pipelines. Nat. Biotechnol..

[42] Love M. I., Huber W., Anders S. (2014). Moderated
estimation of fold change
and dispersion for RNA-seq data with DESeq2. Genome Biol..

[43] Ge S. X., Jung D., Yao R. (2020). ShinyGO: a graphical gene-set enrichment
tool for animals and plants. Bioinformatics.

[44] Wu T., Hu E., Xu S., Chen M., Guo P., Dai Z., Feng T., Zhou L., Tang W., Zhan L. (2021). clusterProfiler
4.0: A universal enrichment tool for interpreting
omics data. Innovation.

[45] Luo W., Friedman M. S., Shedden K., Hankenson K. D., Woolf P. J. (2009). GAGE: generally applicable gene set enrichment for
pathway analysis. BMC Bioinf..

[46] Luo W., Brouwer C. (2013). Pathview: an R/Bioconductor
package for pathway-based
data integration and visualization. Bioinformatics.

[47] Németh M., Janda T., Horváth E., Páldi E., Szalai G. (2002). Exogenous salicylic acid increases polyamine content
but may decrease drought tolerance in maize. Plant Sci..

[48] Pál M., Hamow K., Rahman A., Majláth I., Tajti J., Gondor O. K., Ahres M., Gholizadeh F., Szalai G., Janda T. (2022). Light spectral composition modifies
polyamine metabolism in young wheat plants. Int. J. Mol. Sci..

[49] Gondor O. K., Tajti J., Hamow K., Majláth I., Szalai G., Janda T., Pál M. (2021). Polyamine
metabolism under different light regimes in wheat. Int. J. Mol. Sci..

[50] Nonogaki H. (2014). Seed dormancy
and germination—emerging mechanisms and new hypotheses. Front. Plant Sci..

[51] Spitzer E., Lott J. N. (1980). Thin-section, freeze-fracture, and energy dispersive
x-ray analysis studies of the protein bodies of tomato seeds. Can. J. Bot..

[52] Jimenez-Lopez J. C., Hernandez-Soriano M. C. (2013). Protein bodies in cotyledon cells exhibit differential
patterns of legumin-like proteins mobilization during seedling germinating
states. Am. J. Plant Sci..

[53] Jimenez-Lopez J. C., Zienkiewicz A., Zienkiewicz K., Alché J. D., Rodríguez-García M. I. (2016). Biogenesis
of protein bodies during
legumin accumulation in developing olive (*Olea europaea* L.) seed. Protoplasma.

[54] Faleiro R., Tessmer M. A., Santo
Pereira A. E., Fraceto L. F., Rampasso M. S., Miranda M. T., Pissolato M. D., Cassola F., Ribeiro R. V., Mayer J. L. S. (2025). Assessment
of the impact of biodegradable lignin nanoparticles
encapsulating IAA on tomato development: from seed to fruit. BMC Plant Biol..

[55] Fedenko V. S., Landi M., Shemet S. A. (2022). Metallophenolomics:
a novel integrated
approach to study complexation of plant phenolics with metal/metalloid
ions. Int. J. Mol. Sci..

[56] Mousavi S. R., Galavi M., Rezaei M. (2012). The interaction
of Zn with other
elements in plants: a review. Intl J Agri Crop
Sci..

[57] Gupta A., Bharati R., Kubes J., Popelkova D., Praus L., Yang X., Severova L., Skalicky M., Brestic M. (2024). Zinc oxide nanoparticles application alleviates salinity
stress by modulating plant growth, biochemical attributes and nutrient
homeostasis in Phaseolus vulgaris L. Front.
Plant Sci..

[58] Niekerk L. A., Gokul A., Basson G., Badiwe M., Nkomo M., Klein A., Keyster M. (2024). Heavy metal
stress and mitogen activated
kinase transcription factors in plants: Exploring heavy metal-ROS
influences on plant signalling pathways. Plant,
Cell Environ..

[59] Xue Y., Yan W., Gao Y., Zhang H., Jiang L., Qian X., Cui Z., Zhang C., Liu S., Wang H., Li Z. (2021). Interaction
Effects of Nitrogen Rates and Forms Combined With and Without Zinc
Supply on Plant Growth and Nutrient Uptake in Maize Seedlings. Front. Plant Sci..

[60] Sharma P. N., Tripathi A., Bisht S. S. (1995). Zn requirement
for stomatal opening
in cauliflower. Plant Physiol..

[61] Mattiello E. M., Ruiz H. A., Neves J. C., Ventrella M. C., Araújo W. L. (2015). Zn deficiency affects physiological and anatomical
characteristics in maize leaves. J. Plant Physiol..

[62] Thomine, S. ; Schroeder, J. I. Plant metal transporters with homology to proteins of the NRAMP family. In The NRAMP family, Molecular Biology Intelligence Unit; Cellier, M. ; Gros, P. , Eds.; Andes/Kluwer Series, 2004; pp. 113–121

[63] Krämer U., Talke I. N., Hanikenne M. (2007). Transition metal transport. FEBS
Lett..

[64] Kondak S., Molnár Á., Oláh D., Kolbert Z. (2022). The role of nitric
oxide (NO) in plant responses to disturbed Zn homeostasis. Plant Stress.

[65] Molnár A., Kondak S., Benkő P., Janovszky P., Kovács K., Szőllősi R., Gondor O. K., Oláh D., Gémes K., Galbács G., Janda T. (2022). Limited Zn supply affects nutrient distribution, carbon metabolism
and causes nitro-oxidative stress in sensitive Brassica napus. Environ. Exp. Bot..

[66] Arnaud N., Murgia I., Boucherez J., Briat J. F., Cellier F., Gaymard F. (2006). An iron-induced nitric
oxide burst precedes ubiquitin-dependent
protein degradation for Arabidopsis AtFer1 ferritin gene expression. J. Biol. Chem..

[67] Chungopast S., Duangkhet M., Tajima S., Ma J. F., Nomura M. (2017). Iron-induced
nitric oxide leads to an increase in the expression of ferritin during
the senescence of Lotus japonicus nodules. J.
Plant Physiol..

[68] Chmielowska-Bąk J., Sobieszczuk-Nowicka E., Arasimowicz-Jelonek M. (2025). Nitro-oxidative nucleotide modifications
in plants and associated microorganisms-signaling sensors or stress
symptoms?. J. Exp. Bot..

[69] Faizan M., Faraz A., Yusuf M., Khan S. T., Hayat S. (2018). Zn oxide nanoparticle-mediated
changes in photosynthetic efficiency and antioxidant system of tomato
plants. Photosynthetica.

[70] Ahmed M., Tóth Z., Rizk R., Abdul-Hamid D., Decsi K. (2025). Investigation of Antioxidative
Enzymes and Transcriptomic Analysis
in Response to Foliar Application of Zn Oxide Nanoparticles and Salinity
Stress in Solanum lycopersicum. Agronomy.

[71] Zhang H., Lu L., Zhao X., Zhao S., Gu X., Du W., Wei H., Ji R., Zhao L. (2019). Metabolomics reveals the “invisible”
responses of spinach plants exposed to CeO2 nanoparticles. Environ. Sci. Technol..

[72] Zhang H., Du W., Peralta-Videa J. R., Gardea-Torresdey J. L., White J. C., Keller A., Guo H., Ji R., Zhao L. (2018). Metabolomics reveals how cucumber (Cucumis sativus) reprograms metabolites
to cope with silver ions and silver nanoparticle-induced oxidative
stress. Environ. Sci. Technol..

[73] Sun L., Wang Y., Wang R., Wang R., Zhang P., Ju Q., Xu J. (2020). Physiological,
transcriptomic, and metabolomic analyses
reveal Zn oxide nanoparticles modulate plant growth in tomato. Environ. Sci.: Nano.

[74] Han Y., Liang A., Xu D., Zhang Y., Shi J., Li M., Li M., Liu T., Qi H. (2024). Versatile roles of
trehalose in plant growth and development and responses to abiotic
stress. Veg. Res..

[75] Zhu S., Zhang S., Wang W., Hu N., Shi W. (2025). Transcriptome
Analysis Reveals Candidate Pathways and Genes Involved in Wheat (Triticum
aestivum L.) Response to Zn Deficiency. Biology.

[76] Lin C., Zhang Z., Zhang Z., Long Y., Shen X., Zhang J., Wang Y. (2025). The Role of
Glutathione *S*-Transferase in the Regulation of Plant
Growth, and Responses to
Environmental Stresses. Phyton.

[77] Thiébaut N., Hanikenne M. (2022). Zn deficiency
responses: bridging the gap between Arabidopsis
and dicotyledonous crops. J. Exp. Bot..

[78] Berestovoy M. A., Pavlenko O. S., Goldenkova-Pavlova I.
V. (2020). Plant fatty acid desaturases:
Role in the life of plants and biotechnological potential. Biol. Bull. Rev..

[79] Dong N. Q., Lin H. X. (2021). Contribution of
phenylpropanoid metabolism to plant
development and plant-environment interactions. J. Integr. Plant Biol..

[80] Kolbert Z., Szőllősi R., Rónavári A., Molnár Á. (2022). Nanoforms
of essential metals: from hormetic phytoeffects
to agricultural potential. J. Exp. Bot..

[81] Rogers D. W. (1962). Indirect
Spectrophotometric Determination of Zinc and Cobalt Using Eriochrome
Blue Black R. Anal. Chem..

