# Supplementary material for: Size-Dependent Zinc Delivery and Nanozyme-Mediated Stress Alleviation by ZnO Nanoparticles in Zinc-Deficient Tomato
Source: J Agric Food Chem. 2026 Oct 10;74(41):32067–86. doi: 10.1021/acs.jafc.6c02493 (PMC13693447; doi:10.1021/acs.jafc.6c02493)
Supplement: Supplementary file 1 [file jf6c02493_si_001.pdf]

Supporting Information

Size-Dependent Zinc Delivery and Nanozyme-Mediated Stress Alleviation by ZnO Nanoparticles in Zinc Deficient Tomato

Selahattin Kondak[1]; Dóra Kondak[1]; Tamás Bodor[1,2]; Gábor Fejes[1,2]; Attila Ördög[1]; Andrea Rónavári[3]; Zoltán Kónya[3]; Réka Szőllősi[1]; András Kukri[1]; Attila Gyenesei[4]; Péter Urbán[4]; József Kun[4,5]; Maria Gracheva[6,7]; Ádám Solti[7]; Kinga Benczúr[8]; Magda Pál[8]; Gabriella Szalai[8]; Ildikó Domonkos[9]; Titanilla Szögi[10]; Fernando A. Casian Plaza[11]; Gábor Galbács[11]; Zsuzsanna Kolbert[1*]

[1] Department of Plant Biology, University of Szeged, 6726 Szeged, Hungary

[2] Doctoral School of Biology, Faculty of Science and Informatics, University of Szeged, 6726 Szeged, Hungary

[3] Department of Applied and Environmental Chemistry, University of Szeged, 6720 Szeged Hungary

[4] Hungarian Centre for Genomics and Bioinformatics, Szentágothai Research Centre, University of Pécs, 7624 Pécs, Hungary

[5] Department of Pharmacology and Pharmacotherapy, Medical School University of Pécs, 7624 Pécs, Hungary

[6] Budapest Neutron Centre, Institute for Energy Security and Environmental Safety, HUN-REN Centre for Energy Research, 1121 Budapest, Hungary

[7] Department of Plant Physiology and Molecular Plant Biology, Institute of Biology, ELTE Eötvös Loránd University, 1117 Budapest, Hungary

[8] Agricultural Institute, HUN-REN Centre for Agricultural Research, 2462 Martonvásár, Hungary

[9] Institute of Plant Biology, HUN-REN Biological Research Centre, 6726 Szeged, Hungary

[10] Department of Pathology, Albert Szent-Györgyi Medical School, University of Szeged, 6720 Szeged, Hungary

[11] Department of Molecular and Analytical Chemistry, University of Szeged, 6720 Szeged, Hungary

[*] Corresponding author: ordogne.kolbert.zsuzsanna@szte.hu

Supplementary materials

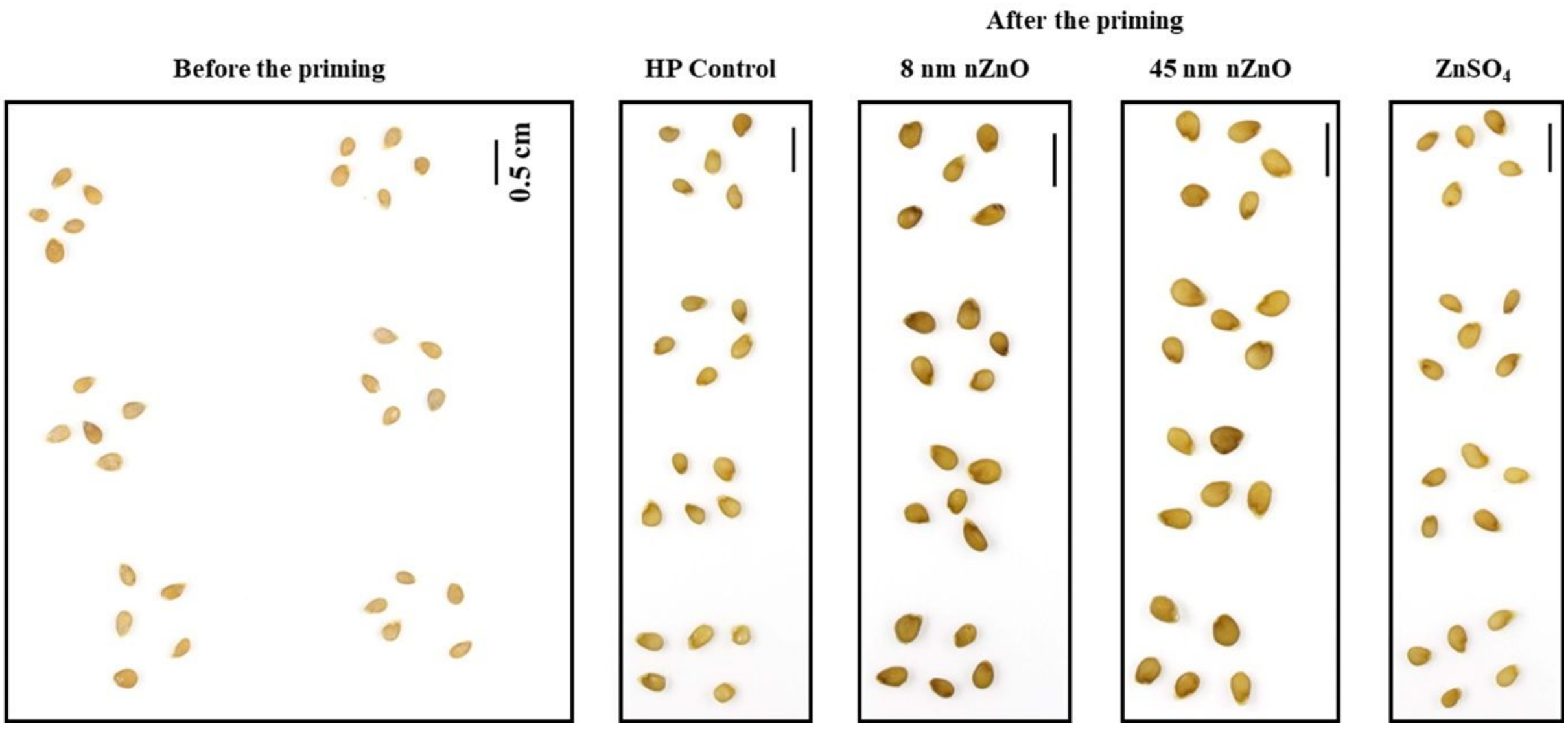

Fig. S1. The appearance of the seeds of tomato before priming and after 24 hours-long priming with distilled water (HP Control), 8 nm nanoZnO (nZnO, 100 mg·L^{-1}), 45 nm nZnO (100 mg·L^{-1}) or $ZnSO_4$ (100 mg·L^{-1}). Scale bar= 0.5 cm.

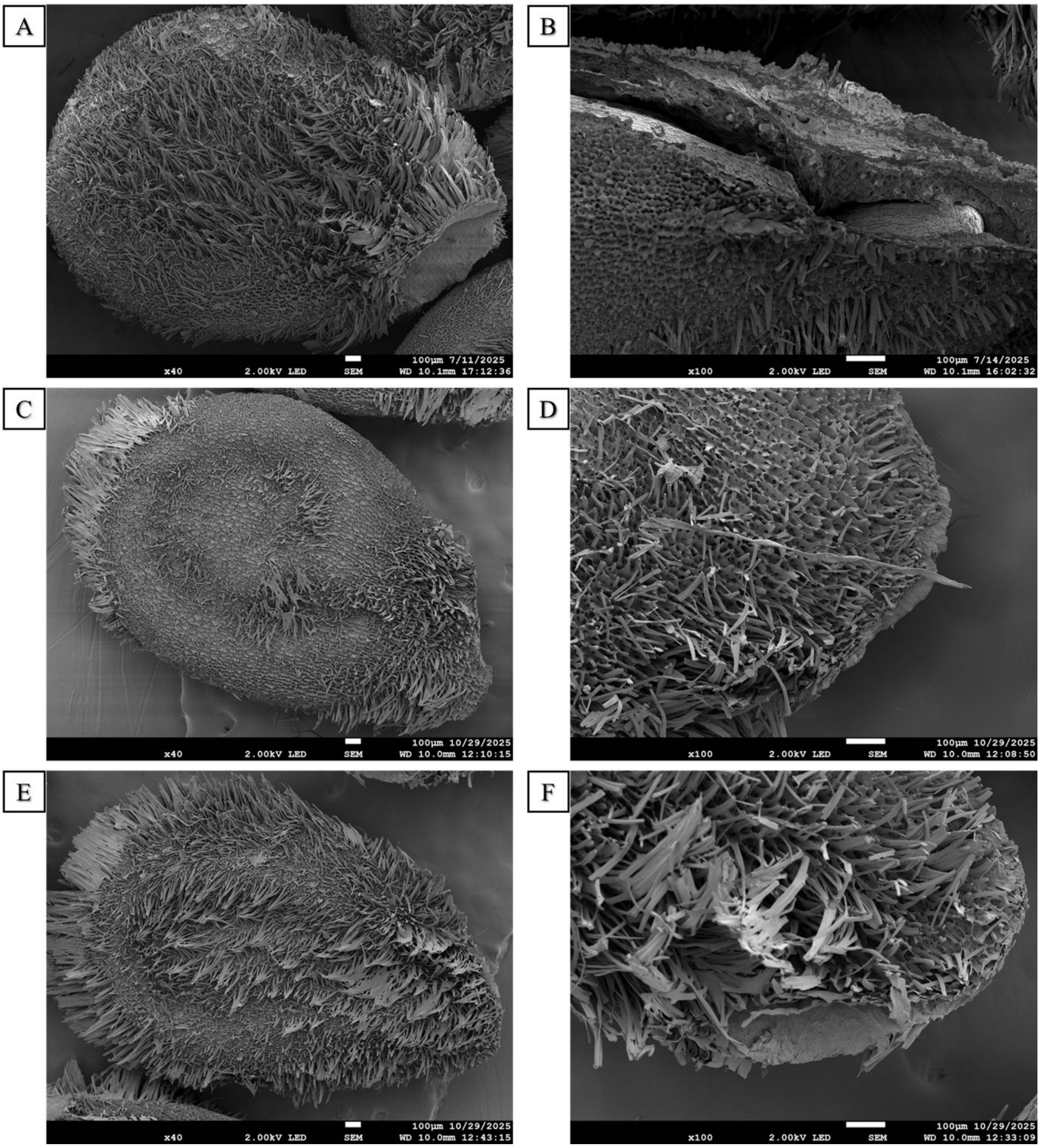

Fig. S2. Scanning electron microscopy (SEM) images of hydroprimed tomato seeds (HP Control) at 40× (A) and 100× (B) magnifications, 8 nZnO-primed of tomato seeds at 40× (C) and 100× (D) magnifications, and 45 nZnO-primed of tomato seeds at 40× (E) and 100× (F) magnifications. Abbreviations: HP= hydroprimed; nZnO= Zn-oxide nanoparticle. Scale bar= 100 µm.

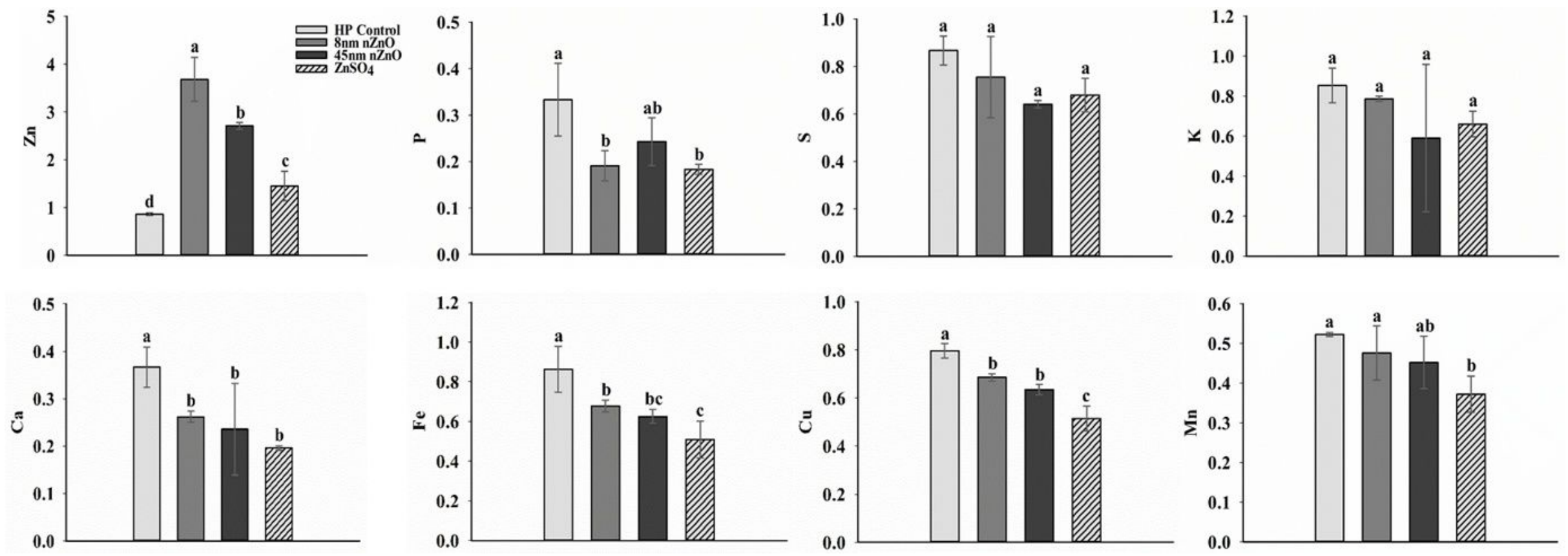

Fig. S3. Normalised contents of different elements measured in inner part of tomato seeds. The seeds were primed for 24 hours with distilled water (HP), 8 nm nanoZnO (nZnO, 100 mg·L^{-1}), 45 nm nZnO (100 mg·L^{-1}) or $ZnSO_4$ (100 mg·L^{-1}). Different letters indicate statistically significant differences according to Duncan's test ($p < 0.05$).

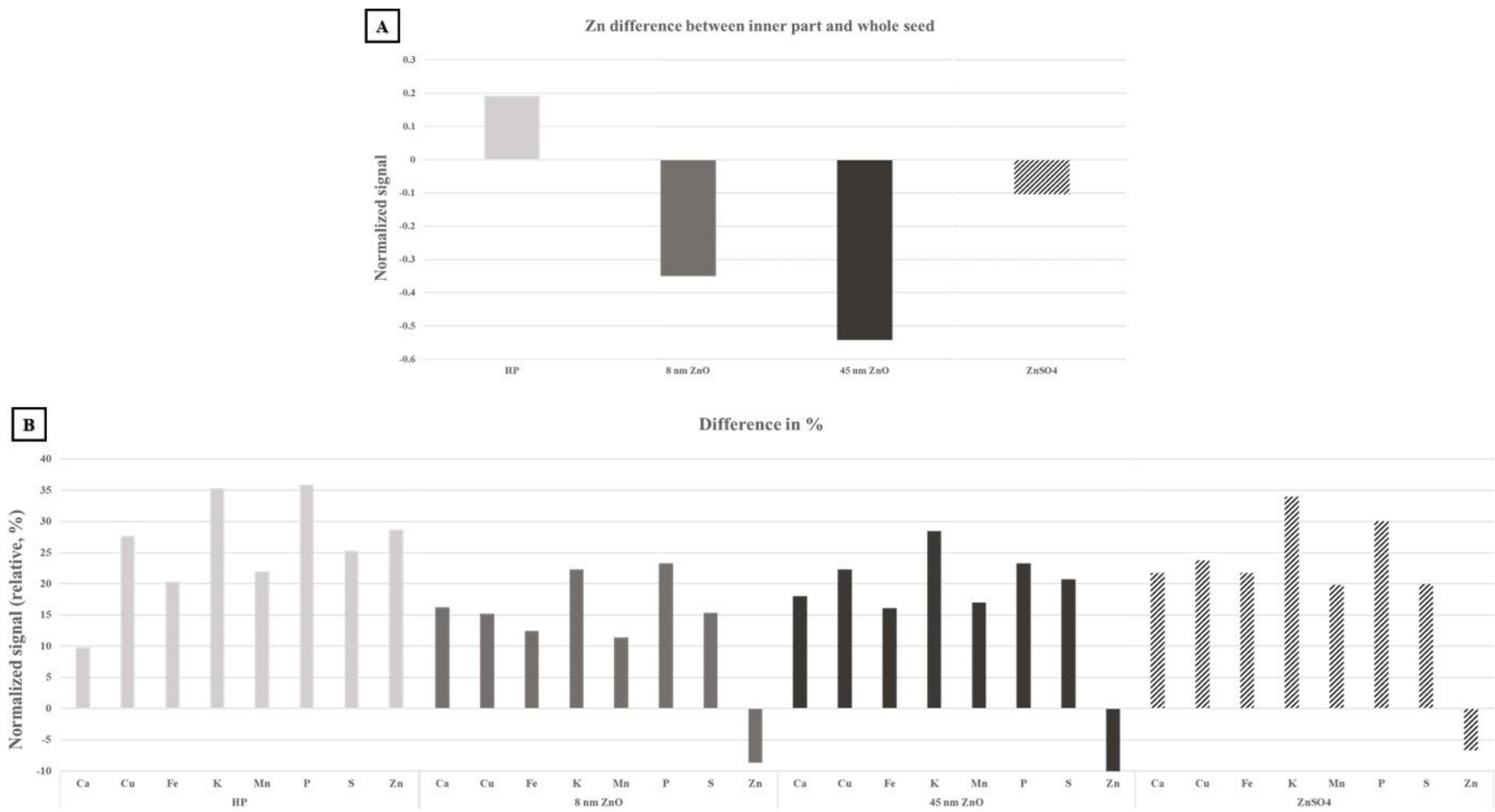

Fig. S4. (A) Difference in normalised content of Zn in inner part of seeds and whole seeds. The seeds were primed for 24 hours with distilled water (HP), 8 nm nanoZnO (nZnO, 100 mg·L^{-1}), 45 nm nZnO (100 mg·L^{-1}) or $ZnSO_4$ (100 mg·L^{-1}). (B) Difference (relative%) in normalized contents of elements in inner part of seeds and whole seeds.

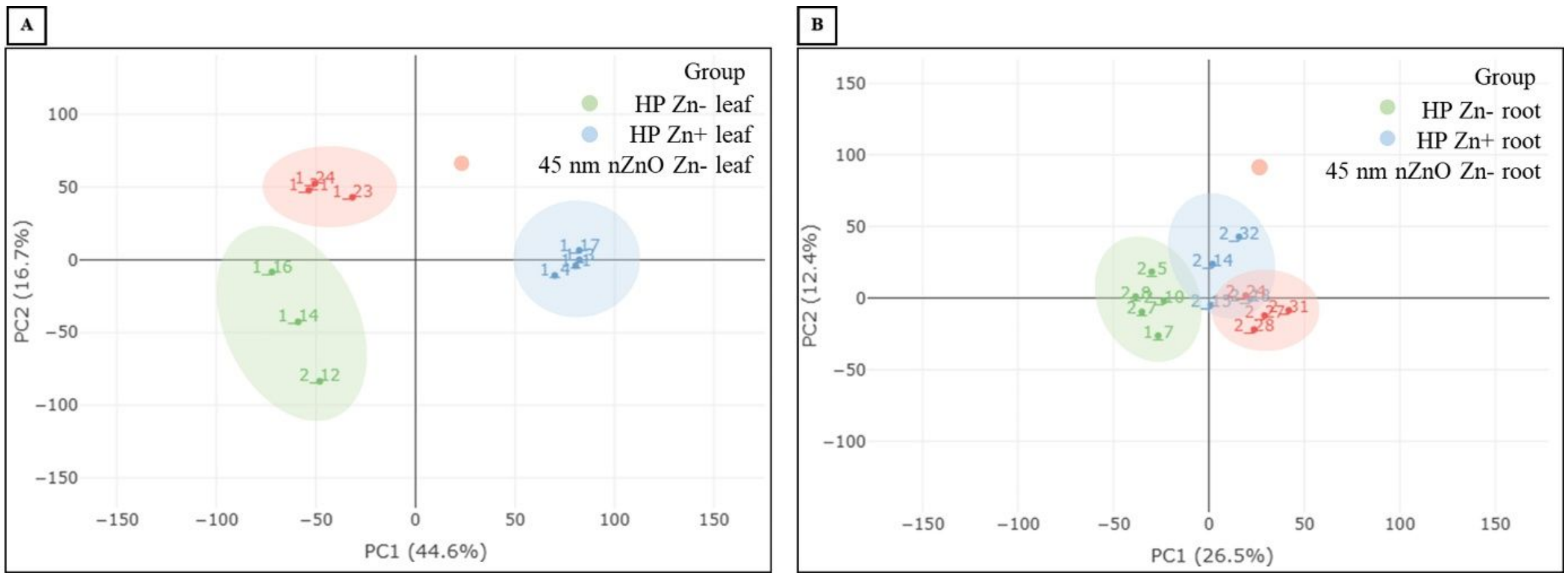

Fig. S5. Plot of results of principal component analysis. Abbreviations: HP= hydroprimed; Zn = Zn-deficient; Zn += Zn sufficient; nZnO= Zn oxide nanoparticle.

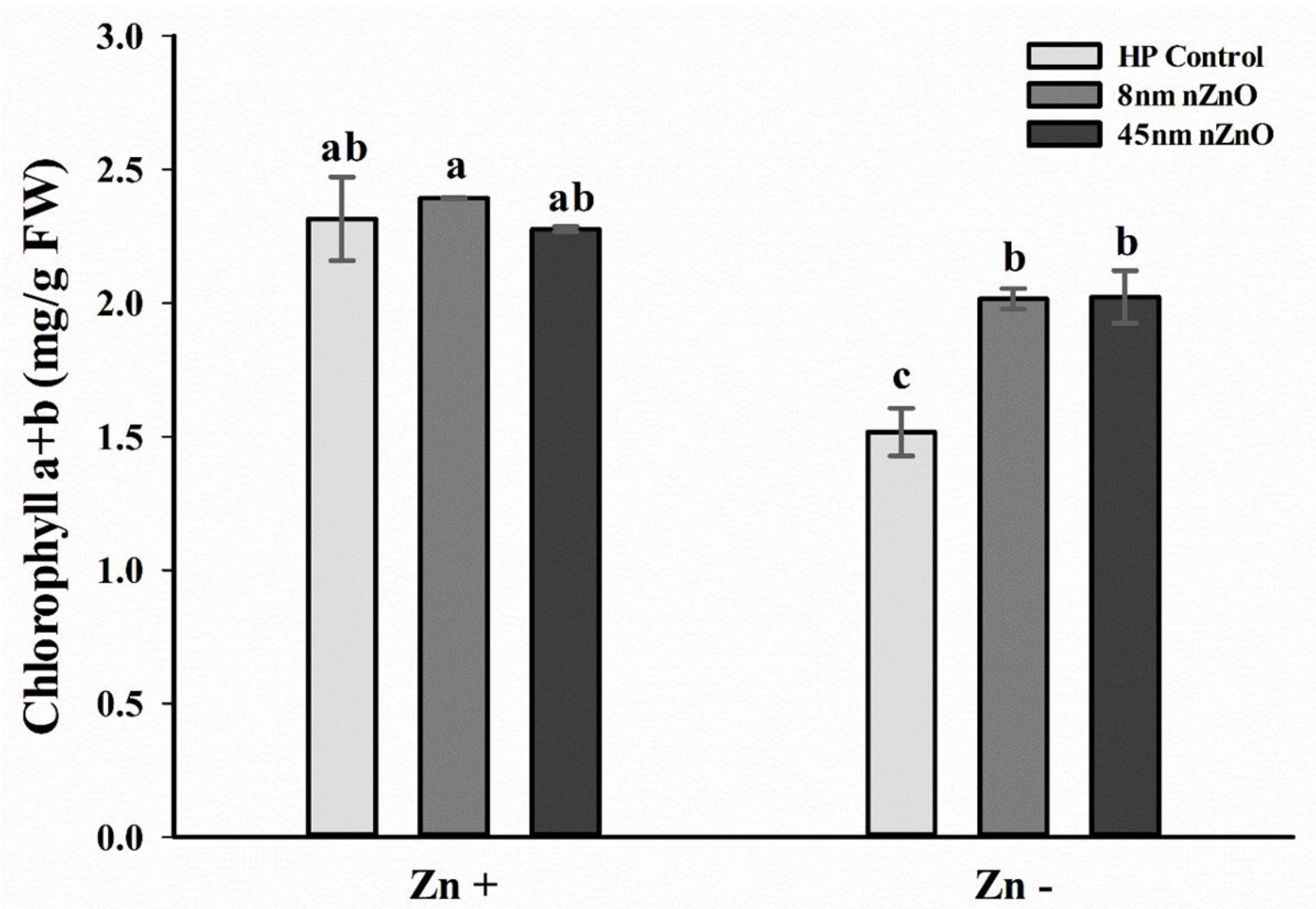

Fig. S6. Total chlorophyll content (mg/g fresh weight) of tomato grown from seeds primed with distilled water (HP Control), 8 nm nanoZnO (nZnO, 100 mg·L^{-1}), 45 nm nZnO (100 mg·L^{-1}) or $ZnSO_4$. Plants were cultivated with optimal Zn supply (Zn +) or with suboptimal Zn supply (Zn -). Different letters indicate statistically significant differences according to Duncan's test ($p < 0.05$).

Table S1. Concentrations (µg/g fresh weight) of amino acids, organic acids and sugars in leaf and root of 3 weeks old tomato grown from seeds primed with distilled water (HP Control), 8 nm nanoZnO (nZnO, 100 mg·L^{-1}) or 45 nm nZnO (100 mg·L^{-1}) and cultivated with optimal Zn supply (Zn +) or suboptimal Zn supply (Zn -). Data are presented as mean ± SD, n= 5.

		Zn (+)			Zn (-)		
	Amino acids µg/g FW	HP Control	8nm nZnO	45nm nZnO	HP Control	8nm nZnO	45nm nZnO
Leaf	L-Valine	33.96 ± 3.26	32.14 ± 4.47	32.74 ± 1.45	54.88 ± 7.88	80.44 ± 25.26	55.36 ± 12.63
	L-Leucine	18.94 ± 5.10	16.86 ± 2.06	15.92 ± 2.54	38.73 ± 7.00	42.68 ± 24.56	34.54 ± 11.75
	L-Serine	110.04 ± 1.35	108.17 ± 2.42	110.63 ± 2.83	140.39 ± 10.81	132.17 ± 12.21	120.21 ± 10.94
	L-Threonine	111.56 ± 1.03	112.49 ± 2.33	112.97 ± 1.62	169.11 ± 29.19	158.80 ± 28.57	136.21 ± 18.97
	Phenylalanine	154.16 ± 4.03	153.38 ± 2.16		232.27 ± 49.77	203.21 ± 15.94	174.60 ± 8.25
	L-Lysine	190.61 ± 1.09	190.62 ± 1.11	191.30 ± 1.32	224.85 ± 23.16	198.86 ± 5.86	209.57 ± 29.27
Root	L-Valine	18.43 ± 0.61	18.35 ± 1.38	17.19 ± 0.94	18.20 ± 1.50	18.55 ± 1.88	20.47 ± 2.06
	L-Leucine	11.85 ± 1.38	11.59 ± 1.82	9.22 ± 3.75	13.44 ± 2.19	10.91 ± 3.76	14.97 ± 2.14
	L-Serine	99.08 ± 0.47	98.01 ± 0.33	98.37 ± 0.60	101.14 ± 1.87	101.38 ± 3.10	106.28 ± 4.06
	L-Threonine	102.38 ± 0.66	102.01 ± 0.66		104.03 ± 1.56	105.73 ± 2.25	111.56 ± 6.92
	L-Lysine	186.26 ± 1.32	185.67 ± 0.64	186.93 ± 0.31	189.14 ± 1.30	188.82 ± 0.94	190.78 ± 0.06
	Organic Acids µg/g FW						
Leaf	Succinic acid	68.57 ± 17.80	61.47 ± 10.53	36.47 ± 2.13	1.17 ± 0.57	35.25 ± 18.87	30.22 ± 9.46
	Fumaric acid	12.68 ± 2.77	29.51 ± 5.60	21.68 ± 7.19	46.97 ± 1.89	15.05 ± 1.05	13.93 ± 3.14
	Malic acid	1728.20 ± 119.20	1762.84 ± 306.29	1873.23 ± 331.23	789.91 ± 137.73	910.56 ± 261.13	1263.85 ± 329.44
	2-Ketoglutaric acid	69.54 ± 2.41	71.27 ± 3.24	65.80 ± 7.81	534.24 ± 66.99	441.27 ± 10.98	886.45 ± 525.91
	Citric acid	941.61 ± 64.36	996.45 ± 176.16	1005.44 ± 197.42	1147.13 ± 488.19	1302.95 ± 261.07	1244.76 ± 219.75
Root	Succinic acid	60.14 ± 10.90	42.24 ± 14.41	47.66 ± 13.48	55.15 ± 22.09	50.88 ± 6.00	66.85 ± 13.68
	Malic acid	407.23 ± 62.62	337.27 ± 88.23	340.80 ± 58.23	405.80 ± 131.48	372.52 ± 16.99	519.59 ± 131.56
	2-Ketoglutaric acid	115.68 ± 17.27	92.26 ± 13.58	98.52 ± 17.15	161.21 ± 20.55	153.35 ± 36.57	147.30 ± 28.70
	Citric acid	89.14 ± 12.75	75.72 ± 7.09	73.17 ± 12.63	281.33 ± 91.15	193.68 ± 25.98	218.35 ± 32.67
	Sugars µg/g FW						
Leaf	Arabinose	39.45 ± 0.95	37.66 ± 1.67	34.09 ± 1.35	32.10 ± 4.71	24.44 ± 3.39	25.05 ± 3.34
	Ribose	50.27 ± 12.04	65.18 ± 3.40	61.71 ± 13.07	56.48 ± 16.69	41.81 ± 15.59	51.37 ± 11.61
	D-Fructose	873.01 ± 114.00	823.09 ± 129.65	910.80 ± 137.55	1168.61 ± 267.07	1154.62 ± 106.05	1016.37 ± 263.63
	D-Glucose	913.91 ± 162.05	838.30 ± 145.84	969.92 ± 160.95	1434.26 ± 363.62	1448.50 ± 254.42	1064.40 ± 416.69
	Galactose	92.14 ± 11.50	89.47 ± 7.66	63.93 ± 25.80	45.97 ± 11.18	15.89 ± 2.81	41.38 ± 19.32
	myo-Inositol	263.64 ± 36.91	249.26 ± 35.30	267.32 ± 45.10	457.22 ± 170.91	421.16 ± 96.04	451.35 ± 74.31
	Cellobiose	51.30 ± 2.72	47.86 ± 4.01	44.29 ± 4.34	59.29 ± 9.88	57.96 ± 6.50	47.51 ± 1.94
	Raffinose	47.91 ± 5.21	58.64 ± 7.09	69.89 ± 4.04	102.40 ± 47.26	91.68 ± 36.42	59.34 ± 25.32
	Maltose	33.43 ± 0.62	33.00 ± 0.43	36.19 ± 4.94	63.43 ± 43.98	53.49 ± 13.38	42.63 ± 9.84
	Trehalose	105.42 ± 13.50	90.95 ± 9.50	74.29 ± 21.06	329.30 ± 97.98	75.68 ± 23.11	76.65 ± 10.78
Root	Arabinose	26.07 ± 0.76	27.17 ± 0.98	26.07 ± 1.84	34.60 ± 1.58	28.13 ± 1.70	27.59 ± 3.12
	Ribose	62.13 ± 1.51	62.80 ± 6.14	61.99 ± 5.11	59.81 ± 20.34	64.94 ± 15.10	65.75 ± 6.15
	D-Fructose	270.18 ± 62.20	241.28 ± 40.25	290.37 ± 91.98	352.48 ± 125.98	422.00 ± 85.28	533.04 ± 25.14
	D-Glucose	269.22 ± 61.85	240.24 ± 39.26	293.06 ± 95.07	356.24 ± 124.57	449.56 ± 117.20	535.95 ± 27.42
	Mannose	5.41 ± 1.00	6.84 ± 1.36	5.41 ± 2.63	8.22 ± 2.70	7.85 ± 0.78	5.58 ± 1.32
	Galactose	17.81 ± 4.66	8.67 ± 1.56	8.60 ± 1.81	24.74 ± 3.72	24.76 ± 2.75	21.32 ± 1.30
	D-Mannitol	21.31 ± 6.82	18.13 ± 3.02	33.50 ± 6.09	47.63 ± 23.27	60.59 ± 14.38	73.36 ± 8.09
	myo-Inositol	21.41 ± 8.29	19.23 ± 7.19	30.35 ± 12.41	91.26 ± 32.65	75.25 ± 11.34	58.19 ± 17.06
	Cellobiose	40.96 ± 1.96	43.25 ± 3.35	42.56 ± 2.01	56.83 ± 2.07	50.45 ± 5.35	44.26 ± 1.83
	Raffinose	31.08 ± 2.79	33.92 ± 1.38	35.03 ± 4.46	42.89 ± 10.44	42.42 ± 5.50	41.91 ± 5.15
	Maltose	32.08 ± 0.68	31.78 ± 1.03	32.32 ± 0.34	34.39 ± 0.95	34.25 ± 0.92	33.17 ± 1.21

Table S2. Concentration (nmol g^{-1} FW) of 1,3-diaminopropane (DAP), putrescine (PUT), spermidine (SPD) and spermine (SPM) in leaves and roots of 3-week-old tomato plants grown from seeds primed with distilled water (HP Control), 8 nm nZnO (100 mg·L^{-1}) or 45 nm nZnO (100 mg·L^{-1}) and cultivated under optimal Zn supply (Zn +) or suboptimal Zn supply (Zn -). Data are presented as mean ± SD, n= 5.

	Polyamine	Zn (+) HP Control	Zn (+) 8nm nZnO	Zn (+) 45nm nZnO	Zn (-) HP Control	Zn (-) 8nm nZnO	Zn (-) 45nm nZnO
Leaf	DAP	0.32 ± 0.05	0.32 ± 0.05	0.33 ± 0.06	0.27 ± 0.04	0.30 ± 0.04	0.29 ± 0.04
	PUT	17.85 ± 5.45	18.42 ± 4.19	20.00 ± 5.51	15.77 ± 4.16	16.64 ± 2.49	18.13 ± 2.43
	SPD	76.00 ± 19.66	76.01 ± 12.62	87.47 ± 20.79	44.66 ± 10.40	46.70 ± 5.09	55.28 ± 8.16
	SPM	25.53 ± 7.38	24.52 ± 4.17	27.96 ± 6.88	14.12 ± 5.47	13.43 ± 2.09	16.22 ± 1.17
Root	DAP	0.68 ± 0.19	0.84 ± 0.37	0.83 ± 0.21	1.83 ± 0.56	2.71 ± 0.70	3.95 ± 1.13
	PUT	9.63 ± 2.02	8.34 ± 1.27	0.83 ± 0.21	16.09 ± 4.28	12.17 ± 0.91	15.02 ± 3.44
	SPD	56.86 ± 6.31	56.59 ± 10.08	60.33 ± 12.26	46.81 ± 7.22	42.67 ± 7.78	54.15 ± 5.37
	SPM	14.54 ± 0.93	15.42 ± 3.00	13.72 ± 1.04	15.83 ± 3.88	15.47 ± 1.04	18.07 ± 2.45

Table S3 Differentially expressed gene classification in leaves (HP Zn + vs. HP Zn -)

Table S3_DEGs
annotated_Cont_Plu

Table S4 Differentially expressed gene classification in roots (HP Zn + vs. HP Zn -)

Table S4_DEGs
annotated_Cont_Plu

Table S5 Differentially expressed gene classification in leaves (nZnO Zn - vs. HP Zn -)

Table S5_DEGs
annotated_45nm_Zr

Table S6 Differentially expressed gene classification in roots (nZnO Zn - vs. HP Zn -)

Table S6_DEGs
annotated_45nm_Zr
